# Advanced Optimization of Clonazepam-Loaded Solid Self-Emulsifying Drug Delivery Systems: Comparison of Weighted Goal Programming and Desirability Function in a Quality by Design Framework

**DOI:** 10.3390/pharmaceutics18030305

**Published:** 2026-02-28

**Authors:** María Luisa González-Rodríguez, Sonia Valverde-Cabeza, Enrique Pérez-Terrón, Antonio María Rabasco, Pedro Luis González-Rodriguez

**Affiliations:** 1 Department of Pharmacy and Pharmaceutical Technology, Faculty of Pharmacy, Universidad de Sevilla, 41012 Sevilla, Spain; sonvalcab@alum.us.es (S.V.-C.); enriqueperezterron@gmail.com (E.P.-T.); amra@us.es (A.M.R.); 2 Department of Industrial Engineering and Management Science, School of Engineering, Universidad de Sevilla, 41092 Sevilla, Spain; pedroluis@us.es

**Keywords:** clonazepam, S-SEDDS, Quality by Design (QbD), Weighted Goal Programming, desirability function, multi-objective optimization, solubility enhancement

## Abstract

**Background/Objectives:** Clonazepam (CLZ), a BCS Class II drug, presents significant oral delivery challenges due to its low aqueous solubility. This study explores the systematic development of solid self-emulsifying drug delivery systems (S-SEDDS) using Quality by Design (QbD). The primary objective was to evaluate and compare advanced mathematical optimization frameworks, specifically Derringer’s Desirability Function (D) and Weighted Goal Programming (WGP), to identify a robust formulation that enhances drug solubilization while ensuring superior processability and flowability. **Methods:** Liquid SEDDS were solidified by adsorption onto a porous matrix (Aerosil^®^ 200/Lactose). A multi-objective optimization was conducted to define a robust Design Space (DS), comparing D against WGP. The trade-offs between competing Critical Quality Attributes (CQAs), specifically powder flowability (angle of repose, AR), blending efficiency (BE), and CLZ recovery (CR), were evaluated. Characterization included morphology from Environmental Scanning Electron Microscopy (ESEM), droplet size analysis, and pH-dependent dissolution studies. **Results:** D provided a highly robust baseline, yielding constant optimal coordinates (F2, F3 = +1; F4 = 0) across all sensitivity levels, with a predicted AR of 40.46°, BE of 0.12 and CR of 90.0%. However, WGP successfully refined this solution by allowing a more flexible weighting of goals, achieving a more favorable compromise with an AR of 38.96°, a BE of 0.11, and a CR of 90.23%. The optimized system maintained nanometric droplet sizes (<200 nm) and showed a controlled, pH-independent release profile, reaching 80% drug solubilization at 6 h. **Conclusions:** Integrating WGP into the QbD framework offers a more versatile and precise optimization than the traditional D for complex pharmaceutical systems. This approach ensures the production of high-quality S-SEDDS, bridging the gap between mathematical modeling and the stringent requirements of industrial solid dosage manufacturing.

## 1. Introduction

Globally, anxiety disorders are among the most prevalent mental health conditions [1,2,3]. While benzodiazepines like Clonazepam (CLZ) remain a cornerstone for acute management due to their potent GABAergic inhibitory effects, acting as positive allosteric modulators of the GABA A receptor [4,5], their long-term use is often limited by side effects and dependence [6]. Recent clinical studies, including meta-analyses, have increasingly supported the anxiolytic efficacy of specific oral lavender preparations (e.g., Silexan^®^), demonstrating comparable effectiveness to low-dose benzodiazepines for generalized anxiety disorder [7], with a favorable safety profile and without risk of dependence or sedation [8]. This suggests a complementary pharmacological approach, since while CLZ acts as a positive allosteric modulator of GABA A receptors, lavender oil modulates voltage-gated calcium channels and the serotonergic system [9], showing minimal competition for the benzodiazepine binding site [10].

However, both CLZ and the active constituents of lavender oil (e.g., linalool) are BCS Class II compounds with poor aqueous solubility, leading to low bioavailability and inconsistent outcomes. To address this, Self-Emulsifying Drug Delivery Systems (SEDDS) have emerged as a robust strategy, spontaneously forming nanoemulsions that increase absorption surface area and bypass hepatic metabolism [11,12,13]. Moreover, to overcome handling and stability issues of liquid forms, solid SEDDS (S-SEDDS) combine these biopharmaceutical advantages with the industrial scalability of solid dosage forms [14,15,16].

The choice of capsules for the delivery of CLZ-loaded S-SEDDS is primarily driven by the requirements of dose uniformity and formulation stability. Given the high potency and low therapeutic dose of CLZ, traditional direct compression or manual filling of powders may present risks of drug segregation and content variability. In contrast, the S-SEDDS approach involves the molecular dispersion of the drug within a lipidic matrix before its adsorption onto a solid carrier, ensuring the active ingredient is uniformly distributed.

While SEDDS/S-SEDDS for CLZ and other lipophilic drugs have been described in the literature to address these challenges [17,18], this study introduces a significant pharmaceutical innovation. Beyond solubilization, this formulation utilizes lavender oil as a bioactive lipidic excipient for a potential combined anxiolytic effect. Within the Quality by Design (QbD) framework, optimizing such complex systems requires balancing multiple conflicting goals [19]. While the Desirability function (D) is a popular tool for finding a compromise score [20,21,22], Weighted Goal Programming (WGP) is less common in this specific context, offering a more flexible approach to handle strict industrial constraints by minimizing deviations from specific targets [20].

To date, a review of the scientific literature has not yielded any articles that employ the WGP methodology for multi-objective optimization of S-SEDDS. Consequently, the objective of this research was to design and characterize an S-SEDDS incorporating CLZ and lavender essential oil, establishing a comparative framework between D and WGP. This study represents a pioneering methodological advancement in the optimization of complex lipid-based drug delivery systems.

## 2. Materials and Methods

### 2.1. Materials

Clonazepam (CLZ) was kindly provided by Prof. F. Maestrelli (University of Florence, Italy). Lipid components for liquid SEDDS included Gelucire^®^ 50/13 and Labrafil^®^ M2130 (Gattefossé, Saint-Priest, France), Polysorbate 80 (Tween^®^ 80; PanReac, Castellar del Vallès, Spain), and vegetable oils (Calendula, Sesame, Wheat germ, Lavender, and isopropyl myristate; Acofarma and Escuder, Terrassa, Spain). Solid carriers for S-SEDDS consisted of lactose monohydrate, microcrystalline cellulose (Roigfarma, Barcelona, Spain), and colloidal silicon dioxide (Aerosil^®^ 200; Acofarma, Spain). Analytical grade reagents for HPLC and dissolution media (acetonitrile, acetic acid, sodium acetate, HCl, NaCl, pepsin, and Na_2_HPO_4_·12H_2_O) were purchased from Labbox (Mataro, Spain), LabKem (Dublin, Ireland), Panreac (Barcelona, Spain), Supelco (Bellefonte, PA, USA), and Sigma-Aldrich (Burlington, MA, USA). Deionized and Milli-Q water were used throughout the study.

### 2.2. Analytical Procedures and Mini-Validation

CLZ was quantified using an HPLC system (Hitachi Elite LaChrom, Tokyo, Japan) with a C-18 column (150 ± 4.6 mm, 5 µm). The mobile phase was a 60:40 (*v*/*v*) mixture of 0.01 M acetic acid/acetate buffer (pH 7) and acetonitrile (flow rate: 0.9 mL/min; injection volume: 20 µL) [21]. Samples were filtered (0.45 µm) before analysis. According to ICH Q2(R1) recommendations, a mini-validation was conducted to confirm method performance within the lipid-based matrix. Specificity was verified by analyzing placebo S-SEDDS, ensuring no chromatographic interference from the surfactants or oil phase. Linearity was confirmed (R^2^ > 0.995) over the concentration range of 10–100 µg/mL. Precision and accuracy were validated with %RSD values <2.0%, and recovery rates within 98–102%, respectively, providing confidence in the drug recovery data obtained during the optimization process.

### 2.3. Development and Characterization of Liquid SEDDS

A qualitative solubility study was conducted to select the vegetable oil of SEDDS, as benzodiazepines are more soluble in oleaginous media [22]. CLZ (1 mg) was mixed with 5 mL of various oils (Calendula, Sesame, Wheat germ, and Isopropyl myristate) at 40 °C for two hours. After selecting the vegetable oil, the optimal concentration of lavender oil for the formulation was determined through a literature review. Based on the technical specifications of a commercial product (Lasea^®^, Karlsruhe, Germany) containing lavender oil as an active ingredient, a concentration of 17% lavender oil was determined to be appropriate for size 0 capsules.

The liquid SEDDS were prepared by varying the proportions of Gelucire^®^ 50/13 (HLB 11), Tween^®^ 80 (HLB 15), Labrafil^®^ M2130 (HLB 9), and Aerosil^®^ 200 (thickener/thixotropic agent). The use of vegetable oils, such as calendula oil, was a strategic choice because studies have shown their positive effects and versatility in self-emulsifying systems, particularly due to their capacity to be efficiently adsorbed onto solid carriers [23]. The formulations are detailed in Table 1.

SEDDS were characterized in terms of droplet size and polydispersity index (PDI) of the emulsions formed upon contact with an aqueous medium simulating gastric condition [23]. Characterization involved adding 1 g of formulation to 100 mL of simulated gastric fluid (USP, pH 1.2 with pepsin) at 37 °C and 500 rpm for two hours. Measurements were performed by Dynamic Light Scattering (DLS) using a Malvern Zetasizer^®^ Nano-ZS 90 (Worcestershire, UK).

### 2.4. Preparation and Characterization of Clonazepam-Loaded Solid SEDD

**Figure 1:**
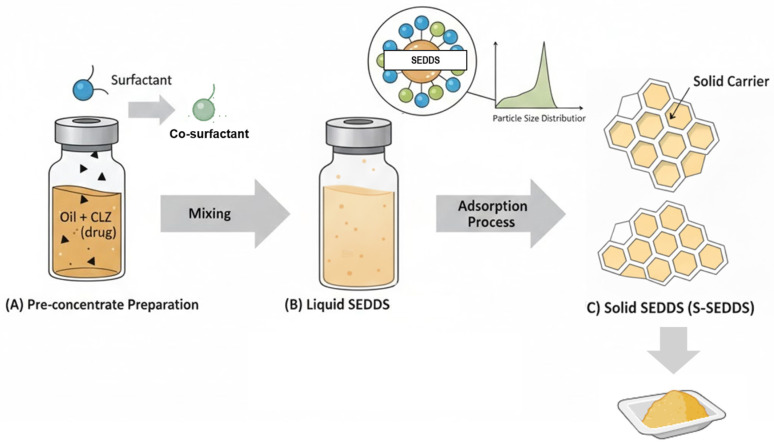
Schematic representation of the S-SEDDS development process. (**A**) Preparation of the lipid pre-concentrate containing CLZ, and the oils (lavender and calendula); (**B**) Preparation and characterization of the liquid SEDDS; and (**C**) Solidification process via adsorption onto a solid carrier matrix to obtain the final free-flowing S-SEDDS powder.

After selecting the most adequate SEDDS formulation, the next step was to choose the most suitable technique to convert the liquid SEDDS into a solid form (S-SEDDS). Among the most common and effective methods for this conversion are lyophilization (freeze-drying), spray-drying, hot-melt extrusion, and adsorption onto solid carriers [23]. This last was the chosen method and the process, illustrated in Figure 1, involves impregnating a porous solid carrier with the liquid SEDDS formulation to produce a free-flowing powder [24].

Firstly, several diluent excipients were screened for adsorbing the SEDDS: lactose monohydrate, cellulose microcrystalline, and colloidal silicon dioxide (Aerosil^®^ 200). After adding a fixed amount of SEDDS, the angle of repose (AR) was calculated as a solid flow measurement. This parameter is a critical quality attribute (CQA) in pharmaceutical manufacturing and is defined as the steepest angle of descent relative to the horizontal plane at which a cone of powder or granular material can be piled without slumping [25,26]. This parameter (α) was calculated from the equation:

(1) $$\alpha =\frac{\arctan h}{0.5\cdot d}$$

where *h* is the height of the cone and *d* is the diameter of the flat base

Amounts of 0.5 g of liquid SEDDS were placed in a mortar with a smooth surface and increasing amounts of the solid carrier were added and mixed slowly with the pestle to determine the adsorption capacity of the solid carrier. The solid carrier capable of adsorbing the liquid SEDDS amount, resulting in a free-flowing nonsticky powder, was chosen for further studies. Prepared powders were left to dry at room temperature and stored in a desiccator until further use.

Once selected, lactose and Aerosil^®^ 200 were used as diluents and adsorbing excipients for further studies. An exhaustive optimization of the blending process was performed, studying the percentage of CLZ recovery (CR) in the mix and blending efficiency (BE) as characterization parameters, in addition to the AR.

The BE of the S-SEDDS was evaluated by assessing the uniformity of CLZ distribution within each batch. The procedure involved taking three aliquots from different locations within each sample to ensure a representative analysis of the entire batch. Next, 5 mL of acetonitrile and the samples were thoroughly shaken to facilitate the extraction of CLZ from the S-SEDDS. Finally, the samples were filtered before being analyzed by HPLC. After the aliquots were quantified in CLZ content, the Coefficient of Variation (CV) was calculated. A lower CV value indicates a more homogeneous blend, signifying that the drug is more uniformly dispersed throughout the batch. Conversely, a higher CV suggests a less uniform mixture, which could lead to inconsistent dosing and affect product quality. This test was crucial for ensuring the reproducibility and reliability of the final product.

The CR assay was performed to assess the homogeneous distribution of CLZ throughout the batches. Three aliquots of 50 mg each were taken from every batch produced and placed in Eppendorf tubes. Next, samples were treated and analyzed for CLZ content similarly than before. The CR was calculated as the percentage of CLZ recovered from the samples relative to the theoretical amount of CLZ corresponding to the prepared batch.

### 2.5. Quality by Design Workflow and Statistical Optimization

The development of CLZ-loaded S-SEDDS was systematically conducted according to QbD principles, integrating risk management and experimental design to ensure a robust formulation.

#### 2.5.1. Risk Assessment and Definition of CQAs

Initially, a Quality Target Product Profile (QTPP) was established to define the desired performance of the oral dosage form. Potential risks were identified using an Ishikawa (fishbone) diagram, followed by the building of a risk assessment matrix.

The risk classification was performed by integrating expert knowledge with a review of the literature. Following the ICH Q9 guideline on Quality Risk Management, the assignment of risk levels (Low, Medium, or High) for each material attribute and process parameter was justified based on preliminary screening results and previously reported data on lipid-based delivery systems [23,27,28]. Factors identified as “High Risk”, such as lipid load and adsorbent ratios, were designated as Critical Process Parameters (CPPs) or Critical Material Attributes (CMAs) and were subsequently evaluated in the experimental design. Based on this analysis, the CQAs selected for monitoring included AR, BE and CR.

#### 2.5.2. Design of Experiments (DoE)

A 3-level Box–Behnken experimental design (BBD) was implemented to investigate the influence of the CPPs and CMAs on the CQAs. The independent variables evaluated were: (F1) CLZ concentration, (F2) Lactose/Aerosil ratio, (F3) SEDDS percentage, and (F4) Blending method. A total of 27 experimental runs, including 3 replicates at the center point, were performed to account for experimental error and ensure the reproducibility of the model.

The code representation of independent and dependent variables is listed in Table 2.

The statistical analysis of the experimental data was conducted using the DOE pack 2000 software and the Statsmodel API from Python 3.12, in order to ensure robust characterization of the formulation and process. Initially, an Analysis of Variance (ANOVA) was performed to statistically validate the significance of the model and individual terms, applying a 95% confidence interval (*p* < 0.05). This was complemented by Analysis of Means (ANOM), which visually identifies the main effects and significant deviations of factor levels from the overall mean. To further elucidate the behavior of the factors, marginal means were calculated and plotted, providing a clear representation of how each independent variable influenced the CQAs. Finally, mathematical regression equations were generated for each response, serving as the predictive foundation for the subsequent WGP stage. To visualize the influence of the formulation factors (F1 to F4) on the CQAs (z_1_: AR, z_2_: BE and z_3_: CR), two-dimensional contour plots were generated using the regression models. In this respect and for visualization purposes, response surfaces involving discrete factors were generated by relaxing their discrete constraints. This approach treated these variables as continuous to better illustrate the underlying trends and interaction phenomena.

#### 2.5.3. Multi-Objective Optimization: Desirability vs. WGP

To define the Design space (DS), two different mathematical strategies were compared to resolve the conflict between high lipid loading and powder processability.

Firstly, Desirability Function approach was used to determine the optimal values for the studied factors. For this, each individual response (yi) was transformed into a dimensionless desirability value (di), ranging from 0 (unacceptable) to 1 (ideal), based on the predefined pharmaceutical constraints for each CQA.

For responses requiring maximization (Larger-The-Better, LTB), such as CR (z_3_), the individual desirability was calculated as:

(2) $$d_{i}\left(z_{i}\right)=\left\{\begin{array}{ccc} 0 & if & z_{i}<L_{i}\\ {\left(\frac{z_{i}-L_{i}}{T_{i}-L_{i}}\right)}^{s} & if & L_{i}\leq z_{i}\leq T_{i}\\ 1.0 & if & z_{i}>T_{i} \end{array}\right.$$

Conversely, for attributes targeted for minimization (Smaller-The-Better, STB), such as AR (z_1_) and BE (z_2_), the function was defined as:

(3) $$d_{i}\left(z_{i}\right)=\left\{\begin{array}{ccc} 1.0 & if & z_{i}<T_{i}\\ {\left(\frac{U_{i}-z_{i}}{U_{i}-T_{i}}\right)}^{s} & if & T_{i}\leq z_{i}\leq U_{i}\\ 0 & if & z_{i}>U_{i} \end{array}\right.$$

where *L_i_* and *U_i_* represent the lower and upper boundaries (anti-ideal points), and *T_i_* is the target value (ideal point). The weight factor (*s*) determines the stringency of the optimization; in this study, s was varied from 1 to 5 to evaluate the robustness of the optimal solution and the sensitivity of the DS to stricter satisfaction criteria.

Giving the importance *w_i_* of each response, Global Desirability (*D*) was then calculated as a weighted pondered geometric mean of these individual scores:

(4) $$D={\left(d_{1}^{w_{1}}\cdot d_{2}^{w_{2}}\cdot \ldots \cdot d_{n}^{w_{n}}\right)}^{1/\sum_{i}w_{i}}$$

This mathematical framework ensures that if any individual response fails to meet the minimum requirements (d_i_ = 0), the overall desirability becomes zero, thus strictly adhering to the QbD principles for pharmaceutical safety and efficacy.

Recently, WGP methodology has been widely described by Valverde-Cabeza et al. (2025). Briefly, the specific phases of the WGP methodology include [20]:

(a) Definition of ideal and anti-ideal points. Using software like LINGO, the best achievable value (the ideal point) for each response was determined. This step also identifies the factor values that yield these optimal results. Conversely, the nadir point, representing the worst achievable values, was also obtained to provide a full range of possible outcomes. To ensure a balanced multi-response optimization, responses were normalized using the ideal and nadir points as boundaries.
(b) Definition of aspiration levels for each response. Aspiration levels (*T_i_*) are the target values that a decision-maker sets for each response. They serve as a practical alternative to calculate the entire Pareto frontier, which is often unfeasible. By defining these targets, the optimization process is guided toward solutions that are meaningful and desirable to the user. In this study, STB was defined (for z_1_ and z_2_), in which the goal was to minimize the response, where any value below the aspiration level is more desirable. LTB aims to maximize the response (such as z_3_), where any value above the aspiration level is more desirable. These targets provide a clear focus for WGP, ensuring that the final solution meets specific and predefined goals.
(c) Relative weights of each response. In this stage, weights (*wi*) were assigned to each response to reflect its priority. The weights were obtained by assigning priority values (*ri*) to each response (with 1 being the most important) and then normalizing them so their sum is 1. This ensures that the most important objectives have the greatest impact on the final solution.
(d) Optimization phase. The final phase optimizes models minimizing weighted deviations from aspiration levels, emphasizing critical objectives.

### 2.6. Quality Control of Optimized Formulation

To verify the predictive capacity of the mathematical model, the optimized CLZ-loaded S-SEDDS (F1: 0.1647%, F2: +1, F3: 28%, F4: Mezint) underwent comprehensive physical and chemical evaluation.

#### 2.6.1. Physicochemical and Morphological Characterization

The surface morphology and microstructure were examined using Environmental Scanning Electron Microscopy (ESEM) (Zeiss EVO, Jena, Germany). Samples were sputter-coated with a 10 nm Gold/Palladium layer and analyzed at 20 kV under high vacuum [29]. Additionally, the crystalline state of the drug within the matrix was evaluated via Differential Scanning Calorimetry (DSC) and Thin-film X-ray diffraction (TF-XRD), as previously described in [30,31,32,33] (see Supplementary Materials in Figures S7 and S8).

#### 2.6.2. Pharmaceutical Performance

The uniformity of mass of the optimized CLZ-loaded S-SEDDS hard capsules was evaluated following the Ph. Eur. 2.9.5 monograph [34]. Twenty capsules were randomly selected from the batch prepared using the optimal coordinates (F1: 0.1647%, F2: C, F3: 28%, F4: Mezint).

The uniformity of CLZ content of the optimized CLZ S-SEDDS capsules was also determined according to the Ph. Eur. 2.9.6 (Test A) [34]. In this case, 10 capsules were randomly selected from the batch produced under optimal conditions. Each capsule was opened, and the contents were quantitatively transferred into a volumetric flask, dissolved in the mobile phase (previously described), and quantified using the HPLC method. The acceptance value (AV) was calculated using the formula: AV = |M − X| + k · s, where *X* is the mean drug content (expressed as a percentage of the label claim), *M* is the reference value (M = X if 98.5% ≤ X ≤ 101.5%), *s* is the standard deviation, and *k* is the acceptability constant (*k* = 2.4 for *n* = 10). According to pharmacopoeial specifications, the batch complies with the test if the calculated AV does not exceed 15.

The spontaneous emulsification efficiency of the optimized CLZ-loaded S-SEDDS was evaluated using a dissolution apparatus (Type II). Six capsules, corresponding to the optimized composition, were added to 500 mL of distilled water maintained at 37 ± 0.5 °C with gentle stirring (50 rpm). The self-emulsification time was recorded as the time required to achieve a clear, homogenous dispersion. Following emulsification, the mean droplet size (z-average) and the PDI were determined by DLS. Samples were analyzed at a 90° scattering angle at 25 °C after appropriate dilution to prevent multiple scattering.

To evaluate the impact of the SEDDS technology on drug solubilization, equilibrium solubility studies were conducted for pure CLZ, the optimized liquid SEDDS, and the optimized S-SEDDS. The studies were performed in three different media: purified water, simulated gastric fluid (0.1 N HCl, pH 1.2), and simulated intestinal fluid (phosphate buffer, pH 6.8). For each medium, amounts of the respective samples containing 1 mg CLZ were accurately weighed and added to test tubes containing 1 mL of the corresponding solvent. To ensure reproducibility and statistical validity, the assay was carried out in triplicate for each condition. The tubes were maintained under constant stirring at 37 ± 0.5 °C for 72 h until equilibrium was reached. After this period, the samples were centrifuged and filtered through a 0.45 µm membrane. The concentration of CLZ in the supernatant was quantified using the chromatographic method.

The drug release profiles of the optimized CLZ-loaded S-SEDDS were evaluated using an Agilent 708-DS USP Dissolution Apparatus 1 (basket method) at 37 ± 0.5 °C and a stirring rate of 50 rpm. To simulate different physiological conditions, the study was conducted in 750 mL of 0.1 N HCl (pH 1.2), followed by 250 mL of 0.2 M Na_2_PO_4_·12H_2_O, and the pH was adjusted to 6.8. HPMC capsules containing the optimized formulation were placed into the dissolution vessels. At predetermined time intervals (30, 60, 90, 120, 180, 240, 300 and 360 min), 5 mL samples were withdrawn, filtered through a 0.45 µm membrane, and analyzed for CLZ content using the HPLC method. To maintain sink conditions, an equal volume of fresh, pre-warmed medium was replaced after each sampling. Powder CLZ was equally added for comparison. The results were expressed as the cumulative percentage of drug released over time.

## 3. Results and Discussion

### 3.1. Selection of SEDDS Ingredients and Formulation Rationale

The solubility screening identified calendula oil as the optimal lipid carrier, providing a clear, homogenous solution for CLZ without evidence of recrystallization. This choice aligns with the known affinity of benzodiazepines for oleaginous media [22]. Furthermore, the incorporation of a fixed 17% (*w*/*w*) standardized pharmaceutical-grade lavender oil in all samples ensures a consistent concentration of key bioactive compounds (such as linalool and linalyl acetate), which would provide superior therapeutic potency and predictable pharmacokinetic behavior. This dual-oil phase (calendula/lavender) acts as a novel natural oily phase that, as suggested by Meirinho et al. (2022), enhances bioavailability by protecting poorly water-soluble drugs within lipid droplets [35]. The inclusion of 3% Aerosil^®^ 200 in the liquid pre-mix provided essential thixotropic properties. According to the literature, this porous silica exhibits high adsorption capacity and prevents droplet coalescence by forming a three-dimensional network through hydrogen bonding, which is critical for maintaining stability prior to solidification [36,37].

As previously described, five SEDDS formulations (F1–F5) were developed by increasing the calendula oil load (0, 8, 18, 28 and 38% *w*/*w*) while maintaining a fixed 17% lavender oil fraction and 3% Aerosil^®^. To accommodate the increasing oil concentration, the surfactant/co-surfactant ratio (Gelucire^®^ 50/13 and Labrafil^®^ M2130) was proportionally reduced (Table 1).

All formulations remained robust in simulated gastric medium (pH 1.2), maintaining CLZ in a solubilized state. However, DLS analysis revealed a bimodal distribution (Figure S1a–e). Population 1 (approx. 74–84% intensity) represents the primary drug-loaded droplets, while Population 2 consists of smaller surfactant-rich mixed micelles (Table S1). This coexistence is a well-documented phenomenon in SEDDS; as the oil-to-surfactant ratio reaches equilibrium, the oil facilitates the curvature of the surfactant interface, refining the dispersion [38].

A significant breakthrough was observed when replacing Gelucire^®^ 50/13 with Tween^®^ 80 (Formula 4 vs. Formula 6). Gelucire^®^, a complex mixture of glycerides and PEG esters, reached a plateau effect where further surfactant increase did not reduce droplet size [39], as shown in Table S1. In contrast, the transition to Tween^®^ 80 induced a dramatic shift, reducing the nanometric population to 46.67 ± 10.64 nm. This is consistent with reports showing that high-HLB non-ionic surfactants (HLB ≈ 15) with flexible polyoxyethylene chains promote higher interfacial curvature and smaller droplets in o/w nanoemulsions [40,41].

The PDI remained between 0.40 and 0.50 across all batches (Table S1). While these values reflect the bimodal nature of the system, the lack of significant variance (*p* > 0.05) indicates that the self-emulsification process is robust. In the nanoemulsion region, such self-assembly leads to thermodynamically robust formulations that resist drug precipitation [42,43]. This behavior suggests that droplet architecture is primarily governed by the interfacial organization of the surfactant rather than the bulk volume of the lipid phase [44,45]. This role of the oil phase as a structural bridge was confirmed by Batch 0 (0% oil), which exhibited the largest PDI and mean size. The addition of lavender and calendula oils promoted more compact structures, justifying the selection of Formula 6 as the most stable formulation. This optimized liquid system, when adsorbed onto hydrophilic carriers like lactose, yields solid systems that redisperse rapidly in gastrointestinal fluids without drug crystallization, fulfilling the primary objective of this pharmaceutical innovation [46,47].

### 3.2. Quality-by-Design Applied to Solid Self-Emulsifying Formulations

To optimize the transformation of liquid SEDDS into solid systems (S-SEDDS), a QbD approach was implemented, starting with the definition of the Quality Target Product Profile (QTPP), as shown in Table 3.

The QTPP established critical attributes such as content uniformity (AV < 15) and Powder Flowability (AR < 40°), which are paramount for high-potency drugs like CLZ to ensure safety and dosing precision in HPMC capsules. In addition, this paper focuses on the behavior of the system upon contact with gastrointestinal fluids. A self-emulsification capacity of <2 min is essential for rapid drug presentation. Furthermore, maintaining a nanometric droplet size (<200 nm) is critical to maximize the surface area, which directly enhances the dissolution rate and bioavailability of poorly water-soluble drugs [35]. Finally, the target of > 80% release within 6 h ensures that CLZ remains available for absorption throughout the intestinal window, preventing the rapid precipitation typically observed with conventional solid forms.

**Figure 2:**
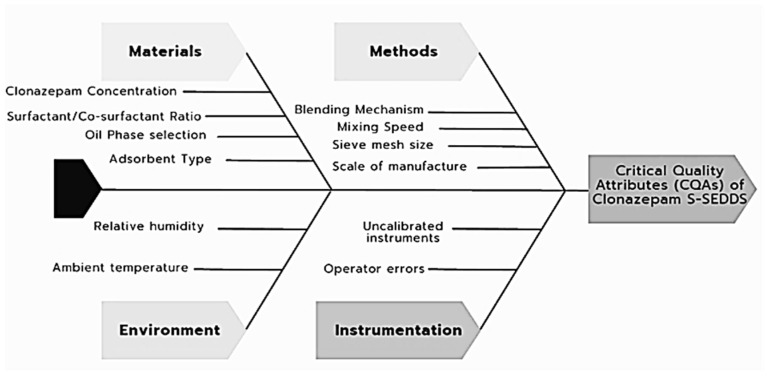
Ishikawa cause-and-effect diagram used for the risk assessment of CLZ-loaded S-SEDDS development under a QbD framework.

The systematic mapping of variables via the Ishikawa diagram (Figure 2) and the Initial Risk Assessment (Table 4) allowed for the prioritization of factors with the highest potential impact on CQAs.

Based on the preliminary identification of variables through the Ishikawa diagram, a risk assessment matrix was developed to prioritize the factors with the highest potential impact on the CQAs. This systematic evaluation utilized a color-coded categorization, where red signifies high risk, yellow moderate risk, and green low risk, to justify the selection of independent variables for the optimization phase. As shown in Table 4, the factors of CLZ concentration (F1), Lactose/Aerosil ratio (F2), percentage of SEDDS (F3), and the blending mechanism (F4) were identified as critical, as they directly influence the flowability of the powder and the homogeneous distribution of CLZ into the S-SEDDS. This proactive prioritization, as highlighted by Buya et al. (2024), is instrumental in identifying that the interaction between the lipid load and the solid carrier surface area represents the most critical uncertainty for the development of functional design spaces [48].

From this comprehensive overview, the four highest-risk factors (CLZ concentration, % SEDDS, Lactose/Aerosil ratio, and Blending mechanism) were isolated for further quantitative study via DoE.

### 3.3. Statistical Analysis and Model Validation

Based on the preliminary risk assessment and the prioritization of formulation factors, a three-level, four-factor BBD (previously described in Section 2.5.2) was employed to systematically investigate the main, indirect, and quadratic effects of the independent variables on the S-SEDDS performance. The experimental results for the three studied responses (AR (z_1_), BE (z_2_), and CR (z_3_)) are summarized in Table 2.

To provide a robust scientific basis for the optimization process, the experimental data were analyzed using a two-stage statistical approach. First, the significance of the factors and their interactions was established through multi-linear regression ANOVA (Tables S2–S4) and subsequently, the direction of these effects was visualized using analysis of means (ANOM) charts (Figures S2–S4). Globally, the One-Way ANOM X-bar visualized in these charts confirmed that the majority of experimental runs remained within statistical decision limits (LDL and UDL), ensuring that observed trends were driven by controlled variables rather than experimental error.

**Figure 3:**
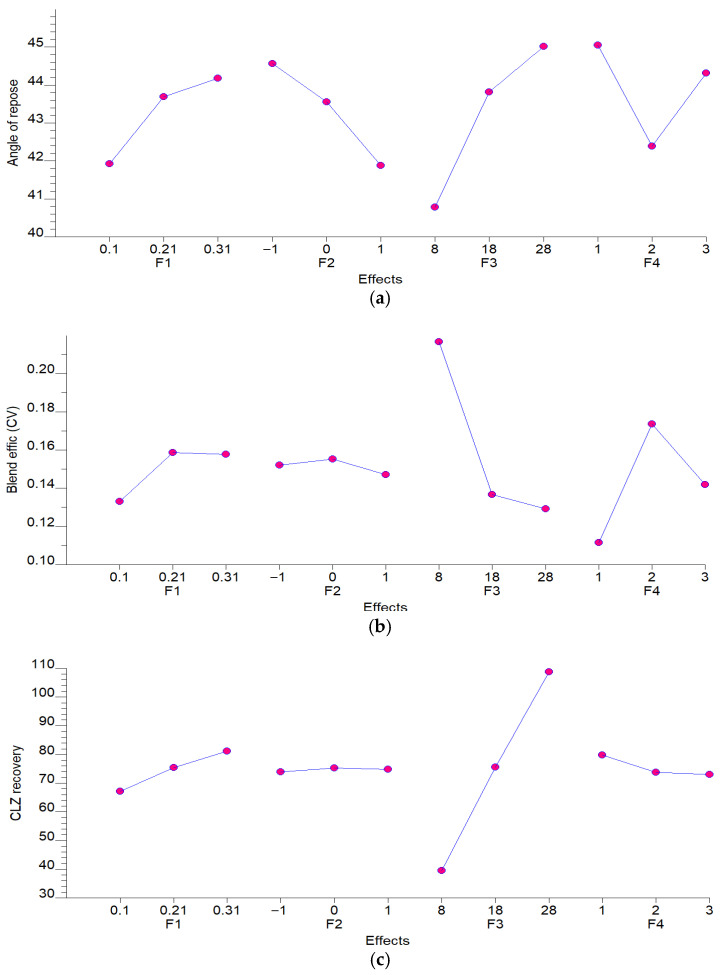
Marginal means plots showing the main effects of independent variables on (**a**) Angle of Repose (z_1_), (**b**) Blending Efficiency (z_2_) and (**c**) CLZ Recovery (z_3_). Each point represents the average response at a specific factor level (F1: [CLZ], F2: Lactose/Aerosil ratio, F3: SEDDS %, and F4: Blending Mechanism). These plots highlight the linear and non-linear sensitivities of the CQAs to the formulation and process parameters.

The simultaneous analysis of ANOVA and ANOM revealed that formulation factors exert distinct and often conflicting influences. A critical trade-off was identified regarding the SEDDS percentage (F3). As shown in the Marginal Means Plots (Figure 3), F3 is the primary driver for drug potency; CR (z_3_) increased dramatically from ~38% at 8% liquid load to over 100% at 28% load (F-value = 247.38). Similarly, for BE (z_2_), which seeks minimization in this response, the increase in SEDDS percentage results in reduced CV values exceeding the decision limits, ranging from 0.21 to 0.13 approx (F-value 10.82). However, this same increase in F3 proved detrimental to physical goals, raising the AR (z_1_) from 40.5° to nearly 45° (F-value: 10.65), shifting the powder from a free-flowing to a cohesive state.

Factor F3 (% SEDDS) follows a conflicting pattern; while certain levels favor the reduction in z_1_ (minimization goal), they simultaneously negatively impact the recovery z_3_ (maximization goal).

From a mechanistic standpoint, this phenomenon is explained by the saturation of the adsorption capacity of the Lactose/Aerosil^®^ 200 system. As the liquid load increases, the oily phase is no longer confined within the pores of the carriers. The excess liquid on the external surface promotes the formation of interparticulate liquid bridges, which significantly increase cohesive forces and internal friction [49,50]. This critical role of the solid carrier in mitigating cohesive effects is consistent with findings by Aloisio et al. (2019) [51].

These results confirm that no single combination of factors can independently satisfy all criteria. While certain levels favor chemical recovery, they inherently compromise physical flowability and binding stability.

The relationship between formulation factors and responses was quantified using polynomial regression equations (Equations (5)–(7)). These equations quantify the magnitude and direction of the main effects, as well as the complexity of the interactions and quadratic behaviors within the S-SEDDS matrix previously identified in the marginal means analysis. Although the BBD primarily supports quadratic architectures, certain higher-order interaction terms were retained in the final model because they significantly contributed to the predictive accuracy (*p* < 0.05) and successfully accounted for the complex physical interactions between the solid carrier and the lipidic components.

(5) $$AR=42.9+1.9\cdot F1-2.3\cdot F2+1.7\cdot F3+0.1\cdot F4-0.3\cdot {F1}^{2}-0.6\cdot {F3}^{2}+2.1\cdot {F4}^{2}-1.0\cdot F1\cdot {F2}^{2}+2.8\cdot {F1}^{2}\cdot F2-1.1\cdot F1\cdot {F3}^{2}+1.2\cdot {F1}^{2}\cdot F3-1.4\cdot {F3}^{2}\cdot F4$$

(6) $$BE=0.150+0.003\cdot F1-0.007\cdot F2-0.020\cdot F3+0.072\cdot {F4-0.082\cdot F1\cdot F3+0.049\cdot F2\cdot F3-0.013\cdot F1\cdot F4-0.030\cdot F3\cdot F4+0.033\cdot F3}^{2}-0.030\cdot {F4}^{2}-0.070\cdot {F2}^{2}\cdot F3-0.135\cdot {F1}^{2}\cdot F4-0.049\cdot {F2}^{2}\cdot F4$$

(7) $$CR=76.4+7.0\cdot F1+34.6\cdot F3-3.4\cdot F4-1.7\cdot {F1}^{2}-1.7\cdot {F3}^{2}$$

The model for AR (z_1_) revealed that F2 has a significant negative effect (−2.3), indicating that increasing this factor improves the flowability of the solid system. Conversely, F1 and F3 exhibited positive coefficients, suggesting a tendency to increase interparticle friction. The presence of higher-order terms, such as F1^2^·F2 and F1·F2^2^, highlights a non-linear relationship, where the impact of the liquid phase on the solid carrier flowability depends strictly on the concentration levels of the other components.

BE (z_2_) was predominantly influenced by F4 (+0.072), identifying it as the primary driver for successful SEDDS incorporation. However, the strong negative interaction between F1 and F3 (−0.082) indicates a competitive effect that could saturate the porous structure of the adsorbent, reducing the overall BE at high concentrations.

The CR (z_3_) was highly sensitive to F3 (+34.6), which showed the largest positive impact among all responses studied. The negative quadratic terms (F1^2^ and F3^2^) suggest a parabolic behavior, indicating that an optimal concentration exists beyond which the recovery efficiency begins to plateau or decrease due to potential physical entrapment or stability limits within the S-SEDDS framework.

Table 5 summarizes the fitting results of response curves, including the mean squared error (MSE) and adjusted values for each response.

Regarding the fitting results, it is noted that the adjusted R^2^ for the AR (z_1_) is relatively lower (~0.52) compared to the other studied responses. This outcome reflects the inherent variability of powder flow properties in S-SEDDS, which are highly sensitive to microstructural interactions and environmental factors that are difficult to fully control experimentally. Despite this, the model successfully captured the directional trends required for the QbD approach, allowing for reliable relative comparisons between formulations. While alternative data transformations and model simplifications were evaluated, they did not yield a significant improvement in predictive power. To mitigate the impact of this inherent uncertainty, AR was strategically treated as a secondary objective during the multi-objective optimization process, ensuring that the final DS decisions were primarily driven by the responses with higher statistical robustness, such as BE and CR.

To comprehensively visualize the multivariate relationships between the independent factors and the CQAs, contour plots were generated based on the established polynomial regression equations and significance of ANOVA (Tables S2–S4). These plots were derived from the response surfaces, illustrating the interaction effects and enabling the identification of regions that satisfy specific quality criteria. 2D contour plots were prioritized in this study against 3D response surface plots to ensure a more precise and rigorous interpretation of the DS coordinates. This choice is primarily dictated by the nature of the experimental design, which involves a combination of continuous (F1 and F3) and discrete factors (F2 and F4). Generating continuous 3D surfaces across categorical transitions is mathematically inconsistent and can lead to misleading interpretations of the physical behavior of the S-SEDDS matrix. Furthermore, given the four-factor complexity of the model, a 3D plot would require fixing two critical factors at arbitrary levels, offering only a partial and potentially biased view of the interactions.

**Figure 4:**
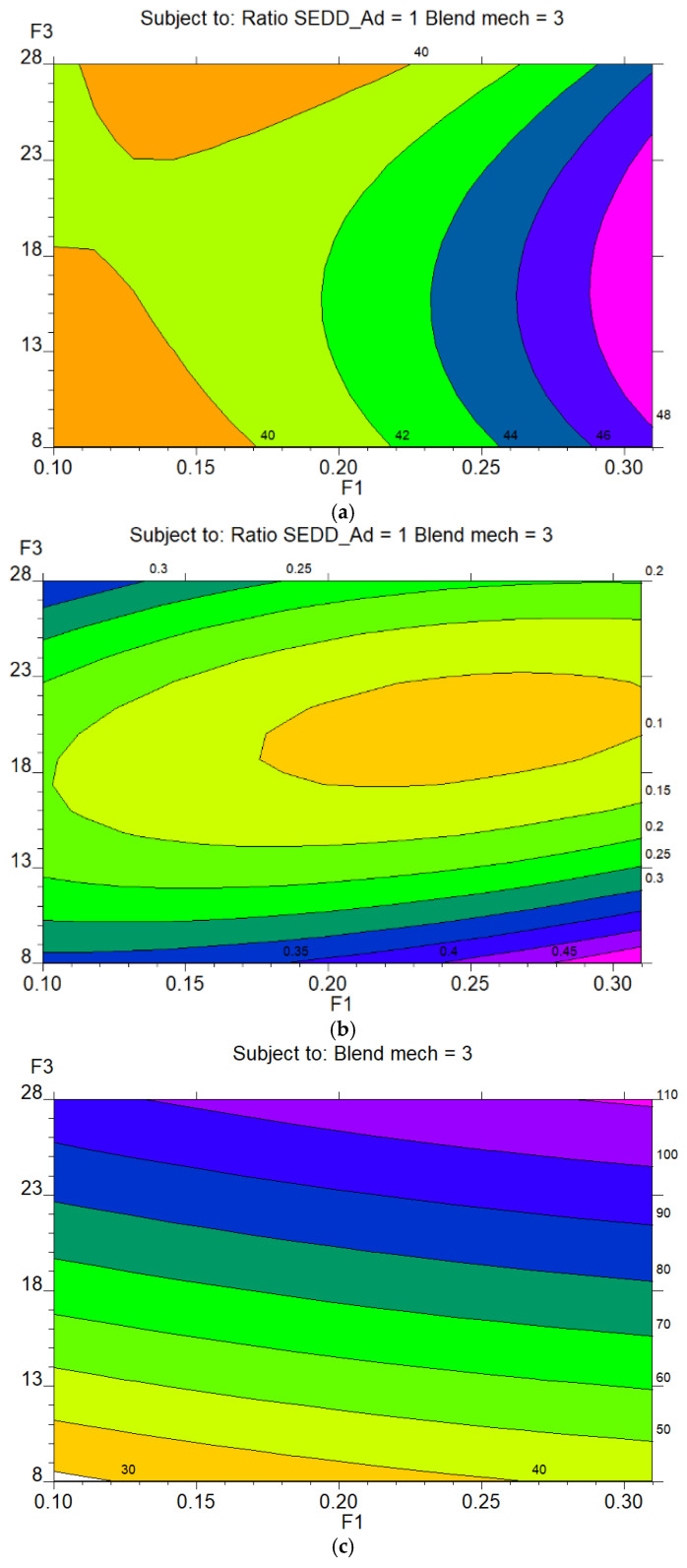
2D contour plots illustrating the interactive effects of independent significant factors (F1: [CLZ] and F3: SEDDS %) on the CQAs. The rows display the response surfaces for (**a**) Angle of Repose (AR, z_1_), (**b**) Blending Efficiency (BE, z_2_), and (**c**) CLZ recovery (CR, z_3_). The color gradients represent the change in response magnitude, where pink to orange-red indicates the transition from higher to lower values. These plots define the operational regions where the pharmaceutical constraints for S-SEDDS are satisfied.

Therefore, the interactions between the continuous factors F1 and F3 versus all CQAs (z_1_, z_2_ and z_3_) (Figure 4) were depicted, as they provide a mathematically continuous representation of the response surface. All other interactions involving the significant discrete factors (F2 and F4) are provided in the Supplementary Material (Figures S5 and S6 for AR and BE, respectively), ensuring a comprehensive yet focused presentation of the multidimensional DS.

The 2D Contour Plots (Figure 4) further illustrate the complexity. Optimal flow (AR < 40°) is achieved at low F1 levels but only within specific and discontinuous intervals of F3 (Figure 4a). Complementarily, the interaction between CLZ concentration (F1) and the Lactose/Aerosil ratio (F2) provides further insight into the system rheology (Figure S5a). As observed in the contour plot, the most favorable flow regions (AR < 40°) are concentrated at higher F2 levels (towards +1.0) and lower F1 concentrations. Finally, the pronounced curvature in the interaction between F2 and F3 indicates that the system operates near a critical saturation point (Figure S5b). Small variations in the oil-to-solid ratio can lead to drastic changes, transitioning the powder from a dry state to a lubricated, cohesive matrix [52].

Regarding the BE plot (Figure 4b), an optimal “homogeneity island” (CV < 0.20) was identified at moderate levels of F1 and F3. The influence of the solid carrier ratio (F2) on mixing efficiency showed a clear trend decrease CV as F2 values were lower, corresponding to a lower proportion of Aerosil in the Lactose/Aerosil binary mixture (Figure S6a,c–e). Furthermore, this response is highly sensitive to F4 (Figure S6b,e,f), according to regression equations, with optimal homogeneity consistently observed when F4 is at its minimum level (−1, manual blending).

The contour plot in Figure 4c illustrates the impact of the factors on CR. Maximum recovery (>95%, depicted in deep blue/purple regions) is predominantly driven by high levels of F3 (23–28%) and F1 (0.21–0.31%). This plot shows a direct correlation, where moving towards higher F3 levels drastically increases drug recovery, emphasizing its critical role in achieving potency.

Collectively, these results highlight a critical conflict between powder flowability and drug recovery, mainly regarding Factor F3. While minimizing the AR and BE favored low levels of F3 (8–15%) or intermediate-higher levels of F3 (18–28%), maximizing CR strictly required high F3 settings (23–28%). Therefore, these contour plots reveal the complex interplay among factors and underscore the presence of conflicting optimal regions for individual CQAs, thus requiring a multi-objective optimization strategy to define a robust DS.

### 3.4. Multi-Objective Optimization and Comparative Analysis

To resolve the inherent conflict between the physical processability (flowability) and pharmacological performance (drug recovery) of the S-SEDDS, a comprehensive multiobjective optimization strategy was applied. This approach integrated two complementary mathematical tools: D and WGP.

#### 3.4.1. Optimization via Desirability Function (D)

The results for the multi-objective optimization using D are summarized in Table 6.

Unlike systems highly sensitive to weight adjustments, this model exhibited remarkable robustness across all evaluated stringency levels (s = 1 to s = 5). The analysis identified a stable “gold standard” combination: F1 at 0.225% (coded 0.19), F2 and F3 at their maximum (+1), and F4 at its central level (0). Although the global D expectedly decreased from 0.860 to 0.469 as satisfaction criteria became more demanding (s = 5), the coordinates remained unchanged. This monotonic decrease is expected because the parameter *s* acts as a curvature (shape) controller in the component desirability functions, making the aggregation more stringent as *s* increases. Because the optimal design remains unchanged, the decrease in D should be interpreted as a change in the scale and strictness of the desirability measure rather than a genuine deterioration of the underlying process performance.

The practical implication is that, once truncation is enforced, the desirability framework becomes dominated by the feasibility of satisfying the “practical acceptability” region (non-zero desirability). In this regime, the optimization is primarily driven by reaching the aspiration thresholds (z_2_ = 0.12 and z_3_ = 90) while maintaining z_1_ within the acceptable range, leading to a unique stable compromise. Consequently, tuning *s* does not provide additional degrees of freedom to explore alternative trade-offs; it only modifies how strongly deviations from the preferred region are penalized in the desirability score.

#### 3.4.2. Optimization via Weighted Goal Programming (WGP)

WGP has emerged as a powerful multi-objective optimization tool in QbD-driven pharmaceutical formulation, enabling balanced optimization of conflicting CQAs in complex lipid-based delivery systems such as ultradeformable liposomes [20].

As a preliminary step, the Ideal and Anti-Ideal points were established using LINGO software (Table 7). These benchmarks provided the mathematical boundaries to normalize the objective functions, aiming for a minimum AR of 35.48°, perfect mixing (BE = 0.0), and maximum recovery of 121.45%.

To locate the optimal compromise within the multi-objective landscape, aspiration levels (*Ti*) and relative weights (*wi*) were assigned to each response based on pharmaceutical quality standards and decision-maker preferences (Table 8).

According to pharmaceutical quality standards, BE and CR were prioritized with a normalized weight of 0.4 each, while AR was treated as a secondary objective (w = 0.2). This weighting strategy was specifically chosen to mitigate the higher statistical uncertainty (R^2^ = 0.52) associated with the flow properties.

These parameters were integrated into a global achievement function (G), which seeks to minimize the weighted sum of unwanted deviations, represented by *p*_1_ and *p*_2_, for the minimization goals, and *n*_3_ for the maximization goal. This mathematical framework ensures that the final solution prioritizes blend homogeneity and CLZ recovery while maintaining acceptable powder flow properties.

Following the described method, the objective function to be optimized for the WGP approach is as follows:

(8) $$minimize\;G=\frac{w_{1}}{T_{1}}\cdot p_{1}+\frac{w_{2}}{T_{2}}\cdot p_{2}+\frac{w_{3}}{T_{3}}\cdot n_{3}$$

The following constraints are considered:

$$z_{i}\left(F\right)+n_{i}-p_{i}=T_{i\;\;\;\;\;}\forall i\left(1,\ldots ,m\right)$$

$$\;\;n_{i},p_{i}\geq 0\;\;\;\;\forall i\;\left(1,\ldots ,m\right)$$

$$F1,\;F3\in \left[-1,1\right]\subseteq R$$

$$F2,F4\in \left(-1,0,1\right)$$

It is important to note that this study considers STB for the first two responses and LTB for the third response. The problem is solved by obtaining the optimal solution using LINGO, where the Global Solver option has been selected. Table 9 shows the details of the solution obtained. Aspiration level is shown as a reference as well as the slacks or deviations (*n_i_* and *p_i_*).

The WGP execution identified the optimal formulation at: F1: 0.1647%, F2: +1, F3: 28%, and F4: +1 (Mezint mechanism). As shown in Table 9, the solution achieved a controlled and quantified deviation of the AR aspiration (38.96° vs 35°), while essentially meeting the targets for BE (0.11) and CR (90.23%).

The deviation variables clearly show how the compromise is achieved relative to the aspiration levels: z_1_ (to be minimized) remains above its aspiration, producing a positive deviation (p_1_ = 3.96), whereas z_2_ is slightly better than its aspiration (since z_2_ < 0.12), which is reflected in a small negative deviation (n_2_ = 0.00592). For z_3_ (to be maximized), the solution marginally exceeds the aspiration level, resulting in a small positive deviation (p_3_ = 0.23). Overall, the WGP solution is characterized by a controlled and explicitly quantified violation of the z_1_ aspiration, while essentially meeting the z_2_ and z_3_ aspirations. This is consistent with the intended behavior of WGP, where trade-offs are mediated through the deviation structure and the relative importance embedded in the goal programming prioritization scheme.

**Figure 5:**
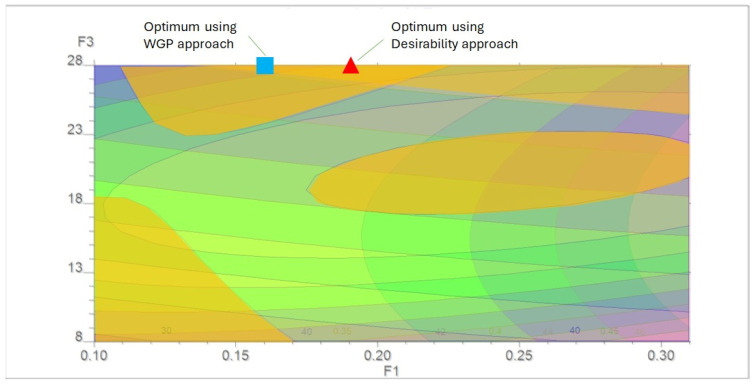
Overlay plot illustrating the multi-response DS as a function of CLZ concentration (F1) and SEDDS percentage (F3). The orange-shaded areas indicate favorable regions for each response. The symbols represent the optimal coordinates identified by the Weighted Goal Programming (WGP) approach (blue square) and the Desirability approach (red triangle), showing their relative positions within the sensitivity regions of the design space.

The multi-objective landscape of the CLZ-loaded S-SEDDS was evaluated by comparing the traditional D with the WGP approach. The overlay plot (Figure 5) serves as a sensitivity map of the DS, illustrating the operational regions where individual CQAs are optimized.

The overlay analysis reveals that the most favorable regions for AR (z_1_), BE (z_2_), and CR (z_3_) are largely disjoint. This spatial separation reflects the inherent trade-offs in multi-response optimization, where maximizing lipid load for drug solubilization adversely affects the physical rheology of the powder. Despite these conflicts, both optimization strategies converge within a similar high F3 slice of the DS, specifically in the region of maximum liquid load (28%).

While both methods identify viable optima, the WGP approach (blue square in Figure 5) identified a coordinate (F1: 0.1647%) distinct from the D approach (red triangle, F1: 0.225%). A direct comparison of the predicted profiles demonstrates the slight but significant superiority of WGP in this study: WGP achieved a lower AR (38.96° vs. 40.46°), provided better BE (0.11 vs. 0.12) and delivered a marginally higher CR (90.23% vs. 90.0%). These results suggest that WGP is better equipped to exploit regions of the DS where all three responses improve simultaneously. Unlike the D method, which relies on arbitrary geometric averaging, WGP provides a transparent, deviation-based resolution. In the D framework, the solution remained fixed at the aspiration thresholds (z_2_ = 0.12, z_3_ = 90%). In contrast, WGP allowed for a controlled and quantified compromise of the ideal AR aspiration to significantly secure the more critical goals.

Recent comparative studies confirm that WGP outperforms traditional desirability functions in complex lipid-based formulations by providing explicit control over response prioritization and avoiding arbitrary geometric averaging of conflicting CQAs [20].

### 3.5. Experimental Verification and Performance Evaluation of the Optimized Formulation

The final stage of the study involved the comprehensive characterization of the CLZ-loaded S-SEDDS prepared under the optimal WGP coordinates (F1: 0.1647%, F2: +1, F3: 28%, F4: Mezint). This formulation was validated against pharmacopoeial and physicochemical standards, as summarized in Table 10.

The optimized batch demonstrated high consistency, complying with Ph. Eur. 2.9.5 for weight uniformity (580 µm ± 3.6 mg) and Ph. Eur. 2.9.6 for content uniformity (AV = 10.3 ± 2.2). This high degree of uniformity is particularly critical for a high-potency drug like CLZ, as it confirms that the S-SEDDS strategy effectively mitigates drug segregation and ensures the dosing precision required for safety. Upon dispersion, the system showed rapid self-emulsification (<120 s), forming a nanometric emulsion with a mean droplet size of 168 nm and a PDI of 0.38. These results align with the growing body of evidence regarding the robustness of S-SEDDS platforms for potent drug delivery [18].

**Figure 6:**
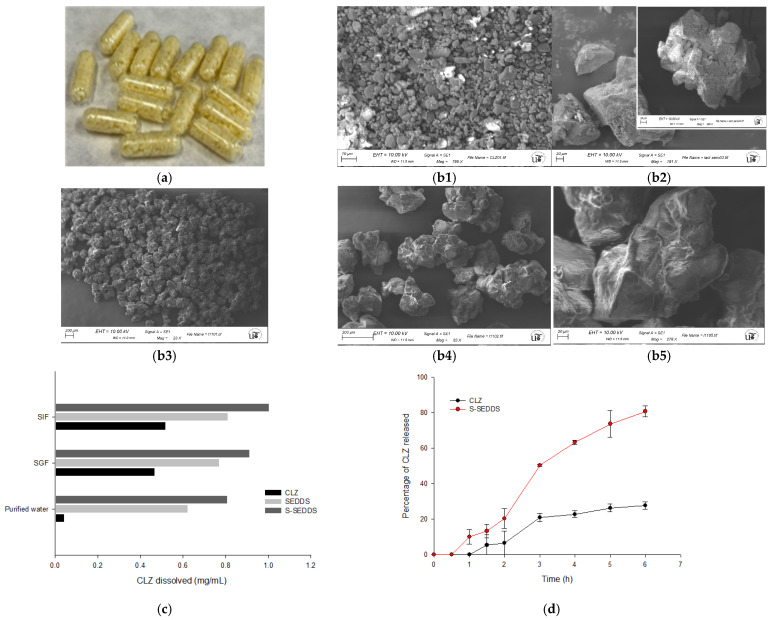
Characterization of the optimized CLZ-loaded S-SEDDS: (**a**) Macroscopic view of the optimized S-SEDDS capsules prepared under the optimal conditions identified by the WGP model. SEM micrographs of: (**b1**) Pure CLZ showing its characteristic crystalline habit; (**b2**) Carrier mixture (Lactose and Aerosil^®^ 200) highlighting the small, spherical Aerosil^®^ nanoparticles adhering to the surface of the larger lactose carriers; (**b3**–**b5**) Optimized S-SEDDS formulation at three magnifications (23×, 83×, and 278×). (**c**) Comparative equilibrium solubility of pure CLZ and SEDDS vs. optimized S-SEDDS in purified water, SGF (pH 1.2), and SIF (pH 6.8). (**d**) Comparative in vitro release profiles of pure CLZ and the optimized CLZ-loaded S-SEDDS. The study was conducted using a sequential pH-change method to simulate gastrointestinal transit: 0–120 min in Simulated Gastric Fluid (SGF, pH 1.2) followed by 120–360 min in Simulated Intestinal Fluid (SIF, pH 6.8). Data are expressed as mean ± SD (*n* = 3).

Macroscopic evaluation (Figure 6a) confirmed well-filled HPMC capsules with no signs of leakage or shell interaction, verifying the suitability of the solid carrier for the high lipid load (28%).

The transformation from liquid to solid was visually evidenced by SEM (Figure 6(b1–b5)). While pure CLZ exhibited a distinct crystalline habit (Figure 6(b1)), these crystals were completely absent in the optimized S-SEDDS micrographs (Figure 6(b3–b5)), indicating that CLZ is either molecularly dispersed or dissolved within the lipid matrix. This transition was further corroborated by DSC analysis (Figure S7). The thermogram of pure CLZ exhibited a sharp endothermic melting peak at approximately 239 °C, confirming its crystalline nature. Similarly, the physical mixtures of the drug with the carriers maintained this characteristic signal. However, in the optimized S-SEDDS formulation, the CLZ melting endotherm completely disappeared, leaving only the thermal events associated with the carrier matrix, such as the lactose dehydration peaks between 140 and 150 °C. The absence of the drug melting peak, in alignment with the SEM observations, provides evidence that CLZ is no longer in a crystalline state but has been successfully transformed into an amorphous form or a molecularly dispersed solid solution within the lipidic film adsorbed on the porous network. The amorphous nature of the drug within the optimized S-SEDDS was further corroborated by XRPD analysis. As shown in Figure S8, pure CLZ exhibited a highly crystalline profile with intense, sharp Bragg reflections, particularly at 2θ values of approximately 12.8°, 15.6°, 20.1°, and 23.3°, resulting in a calculated crystallinity of 89.8%. In contrast, while the S-SEDDS formulation retains certain peaks associated with the crystalline lactose carrier, there is a notable reduction in the overall crystallinity of the system (80.6%) compared to the physical mixture of the drug and carriers (Lact-Ae-CLZ, 89.0%). The disappearance of the specific diffraction signals of CLZ in the S-SEDDS pattern, along with the observed reduction in crystalline intensity, confirms that the drug is successfully integrated into the lipidic-carrier matrix. This structural transformation, in total agreement with the DSC and SEM findings, explains the significantly enhanced dissolution and pH-independent release observed. This structural change is critical, enhancing the biopharmaceutical performance of the system.

The binary carrier mixture (Figure 6(b2)) displayed a high-surface-area architecture where small, spherical Aerosil^®^ 200 microparticles act as a physical barrier, “dry-coating” the larger lactose grains. This microstructural arrangement serves a dual purpose since it preserves powder flowability under lipid loads and facilitates rapid reconstitution. Upon dispersion, the system achieved spontaneous emulsification in 95 ± 11.7 s, forming a nanometric emulsion (168 ± 9.7 nm; PDI 0.38) that satisfies all QTPP requirements.

Equilibrium solubility studies (Figure 6c) revealed that the S-SEDDS significantly increased the concentration of CLZ in all tested media compared to the pure drug, specifically increasing from 0.04, 0.47 and 0.52 mg/mL of CLZ in purified water, SGF and SIF, respectively, to 0.81, 0.91 and 1.00 mg/mL of CLZ-loaded S-SEDDS.

The in vitro release profiles (Figure 6d) further highlighted the efficiency of the lipid-based delivery system. While pure CLZ dissolution remained below 30%, the optimized S-SEDDS exhibited a progressive and sustained release, reaching 80% at 6 h. Unlike many systems that show a burst effect, this formulation showed a controlled solubilization profile. This behavior is fundamentally linked to the internal structure of the matrix [28]; the SEDDS is deeply adsorbed within the capillary channels of the Lactose/Aerosil^®^ 200 network. The droplets must diffuse through the tortuous path of the carrier, which prevents chaotic initial release and local saturation [52].

Critically, the profile remained stable during the pH transition from 1.2 to 6.8 (Figure 6d). For BCS Class II drugs, an increase in pH typically triggers recrystallization, but the S-SEDDS maintained a stable plateau [53]. This robustness suggests that the surfactant-oil droplets effectively shield CLZ molecules from the external environment, preventing nucleation. This pH-independent performance indicates that the drug will remain in a solubilized, absorbable state throughout the gastrointestinal transit, potentially improving the safety and efficacy profile of the treatment [54].

The implementation of the S-SEDDS strategy for CLZ serves a dual purpose. From a technological standpoint, it ensures a highly precise distribution of the API within the lipidic matrix, as evidenced by the excellent content uniformity. From a therapeutic perspective, the formation of a fine emulsion upon contact with gastric fluids is expected to maximize oral bioavailability. This enhancement in solubility and absorption efficiency suggests that the S-SEDDS platform could facilitate dose reduction in long-term treatments, thereby improving the safety profile of the benzodiazepine. The results in Table 10 validate that the optimized formulation adheres to all pharmacopoeial standards required to transition this hypothesis into future in vivo evaluations.

## 4. Conclusions

This study demonstrates that the development of complex lipid-based systems, such as CLZ-loaded S-SEDDS, requires optimization tools capable of handling inherent pharmaceutical trade-offs. The integration of QbD with WGP successfully navigated the resolution of technical conflicts, identifying an optimal coordinate that traditional geometric averaging could not exploit effectively.

This approach provided a transparent, deviation-based explanation of the trade-offs, allowing for a controlled violation of flowability targets to prioritize more critical safety attributes like content uniformity and potency.

The WGP-LINGO platform represents a robust and scalable template for the QbD development of next-generation nanocarriers. By providing explicit control over response prioritization, this methodology streamlines the transition from laboratory-scale formulation to predictable, high-quality industrial manufacturing of complex drug delivery systems.

Regarding the future scope of this research, the promising in vitro results provide a solid scientific basis for the transition to future in vivo evaluations using animal models, which will be essential to correlate the enhanced solubility and the nanometric droplet size with the oral bioavailability and pharmacodynamics of the optimized S-SEDDS. These studies will aim to confirm the potential for dose reduction and the therapeutic synergy with lavender oil, ultimately establishing this WGP-optimized platform as a safer and more effective alternative for long-term benzodiazepine therapy.

## Figures and Tables

**Table 1:** Composition of excipients for SEDDS preparation. CLZ amount was 0.1% *w*/*w*.

	Lavender Oil (%)	Calendula Oil (%)	Gelucire^®^ 50/13 (%)	Labrafil^®^ M2130 (%)	Aerosil^®^ 200 (%)	Tween^®^ 80 (%)
F1	17	0	41.5	38.5	3	-
F2	17	8	37.5	34.5	3	-
F3	17	18	32.5	29.5	3	-
F4	17	28	27.5	24.5	3	-
F5	17	38	22.5	19.5	3	-
F6	17	28	0	24.5	3	27.5

**Table 2:** Experimental matrix of the Box–Behnken design (BBD) for the development of Clonazepam S-SEDDS (top) and definition of independent variables and levels (bottom).

Cod	Coded Values						Outputs		
	F1	F2	F3	F4			z_1_ AR	z_2_ BE	z_3_ CR
R1	−1	−1	0	0			41.1 ± 1.5	0.07 ± 0.02	72.6 ± 3.8
R2	−1	1	0	0			40.6 ± 0.7	0.16 ± 0.05	68.2 ± 8.1
R3	1	−1	0	0			41.2 ± 2.0	0.19 ± 0.01	69.1 ± 8.5
R4	1	1	0	0			43.8 ± 2.9	0.12 ± 0.01	85.9 ± 19.1
R5	0	0	−1	−1			41.3 ± 0.6	0.07 ± 0.01	54.9 ± 3.2
R6	0	0	−1	1			41.5 ± 0.9	0.28 ± 0.03	39.3 ± 11.5
R7	0	0	1	−1			48.7 ± 1.1	0.09 ± 0.02	104.2 ± 11.8
R8	0	0	1	1			43.1 ± 2.9	0.18 ± 0.02	109.2 ± 13.7
R9	0	0	0	0			38.8 ± 1.5	0.21 ± 0.01	58.8 ± 5.1
R10	−1	0	0	−1			44.7 ± 1.4	0.20 ± 0.00	54.3 ± 9.6
R11	−1	0	0	1			38.5 ± 1.5	0.10 ± 0.02	63.8 ± 3.6
R12	1	0	0	−1			41.6 ± 3.0	0.18 ± 0.04	91.1 ± 14.9
R13	1	0	0	1			49.0 ± 2.6	0.03 ± 0.00	100.2 ± 1.5
R14	0	−1	−1	0			41.0 ± 1.1	0.31 ± 0.06	45.0 ± 12.7
R15	0	−1	1	0			45.9 ± 2.0	0.03 ± 0.01	109.9 ± 4.3
R16	0	1	−1	0			38.8 ± 2.6	0.25 ± 0.04	39.5 ± 6.9
R17	0	1	1	0			38.5 ± 2.0	0.17 ± 0.02	103.1 ± 11.8
R18	0	0	0	0			45.8 ± 0.0	0.18 ± 0.01	97.0 ± 9.3
R19	−1	0	−1	0			39.3 ± 3.3	0.10 ± 0.06	28.3 ± 4.1
R20	−1	0	1	0			47.2 ± 0.4	0.22 ± 0.02	115.8 ± 15.0
R21	1	0	−1	0			42.8 ± 1.2	0.29 ± 0.03	30.5 ± 4.3
R22	1	0	1	0			46.6 ± 2.4	0.08 ± 0.02	110.4 ± 4.4
R23	0	−1	0	−1			48.9 ± 0.9	0.13 ± 0.01	87.1 ± 6.6
R24	0	−1	0	1			49.2 ± 0.2	0.18 ± 0.01	60.3 ± 7.8
R25	0	1	0	−1			45.1 ± 1.7	0.04 ± 0.00	87.5 ± 2.2
R26	0	1	0	1			44.5 ± 1.3	0.09 ± 0.02	64.9 ± 0.4
R27	0	0	0	0			44.3 ± 0.2	0.13 ± 0.01	71.9 ± 7.6
Independent variables				Levels			Dependent variables	Goal for dependent variables	
				−1	0	+1			
CLZ concentration (F1, %)				0.1	0.21	0.31	Angle of repose (z_1_)		Minimize
Lactose/Aerosil ratio (F2)				A	B	C	Blending efficiency (z_2_)		Minimize
Percentage of SEDDS (F3, %)				8	18	28	CLZ recovery (z_3_)		Maximize
Blending method (F4)				Bman	Bcon	Bint			

Note: F1: CLZ concentration; F2: Lactose/Aerosil ratio; F3: SEDDS percentage; F4: Blending method. Adsorbent ratios (Lactose/Aerosil^®^ 200) correspond to: A (99.9/0.1%), B (99.5/0.5%), and C (99/1%). Abbreviations: Bcon: continuous blending; Bman: manual blending; Bint: blending by intervals. AR: Angle of Repose; BE: Binding Efficiency; and CR: Clonazepam Recovery.

**Table 3:** Quality Target Product Profile (QTPP) for CLZ-loaded S-SEDDS, including critical attributes, their justification based on patient impact, and predefined target values for pharmaceutical specifications.

Critical Pillar	Key QTPP Attribute	Impact on the Patient/Justification	General Target Value
1. Dosing	Content Uniformity	Ensures each capsule contains the precise dose, critical for high-potency drugs like CLZ.	Complies with Ph. Eur. 2.9.6 (Acceptance Value < 15).
	Powder Flowability	Necessary for consistent volumetric filling during encapsulation.	Angle of Repose < 40° (Fair to Good flow according to Ph. Eur. 2.9.36).
2. Performance	Self-emulsification capacity	Essential for rapid solubilization and presentation of CLZ for absorption in the GI tract.	Spontaneous emulsification in <2 min.
	Nanometric droplet size	Maximizes surface area to increase bioavailability and ensure a rapid onset of action.	Mean droplet size (Z-average) < 200 nm; PDI < 0.4.
	Dissolution rate	Ensures the drug is available for absorption within the absorption window.	>80% release within 6 h in SIF

**Table 4:** Initial risk assessment matrix for the CLZ-loaded S-SEDDS formulation. The table evaluates the potential impact of CPPs and CMAs on CQAs, serving as the basis for selecting the variables studied in the DoE. Risk levels are color-coded as follows: red (high risk), yellow (moderate risk), and green (low risk), representing the prioritization of variables for the optimization phase.

Process Factor	Droplet Size	Flowability	Blending Index	Drug Loading	Overall Risk
CLZ concentration					High
Lactose/Aerosil ratio					High
% of SEDDS					High
Blending mechanism					High
Mixing speed					Medium
Relative humidity					Medium
Adsorbent type					Medium

**Table 5:** Summary of fitting results of the regression curves. AR: angle of repose; BE: blending efficiency; CR: CLZ recovery.

Response	Alias	Criterion	MSE	R^2^_adj_
AR	z_1_	Minimize	5.062464	0.520254
BE	z_2_	Minimize	0.00155	0.71281
CR	z_3_	Maximize	161.2369	0.761573

**Table 6:** Optimization results using Desirability Function (D): comparison of predicted responses (z) and factor levels (F) at different sensitivity (s) settings.

s	z_1_	z_2_	z_3_	D	F1	F2	F3	F4
1	40.46	0.12	90.0	0.860	0.19	+1	+1	0
2	40.46	0.12	90.0	0.739	0.19	+1	+1	0
3	40.46	0.12	90.0	0.635	0.19	+1	+1	0
4	40.46	0.12	90.0	0.546	0.19	+1	+1	0
5	40.46	0.12	90.0	0.469	0.19	+1	+1	0

**Table 7:** Trade-off matrix for individual optimization through LINGO. z_1_: angle of repose; z_2_: blending efficiency; z_3_: CLZ recovery. F1: [CLZ]; F2: Lactose/Aerosil ratio; F3: SEDDS %; F4: Blending mechanism.

Response	z_1_	z_2_	z_3_	F1	F2	F3	F4
Min z1	35.48	0.4811	23.829	1	−1	−1.0	−1
Min z2	42.36	0.0	109.056	0.5789	1	1.0	1
Max z3	45.29	0.1810	121.454	1	1	1.0	−1
Ideal Point	35.48	0.0	121.454				
Anti-Ideal Point	45.29	0.4811	23.829				

**Table 8:** Aspiration levels and decision preferences. AR: angle of repose (z_1_); BE: blending efficiency (z_2_); CR: CLZ recovery (z_3_).

Response	Aspiration Form	Aspiration Level (*T_i_*)	Deviation Variable	Importance (*r_i_*)	Weight (*w_i_*)
AR	z_1_≤	35	p_1_	2	0.2
BE	z_2_≤	0.12	p_2_	1	0.4
CR	z_3_≥	90	n_3_	1	0.4

**Table 10:** Physicochemical and pharmacopoeial characterization of the optimized CLZ-loaded S-SEDDS.

Parameter	Method	Result (Mean ± SD)	Ph. Eur. Specification
Weight uniformity	Ph. Eur. 2.9.5	580 ± 3.6 mg	Max. ±5% deviation
Content uniformity	Ph. Eur. 2.9.6	AV = 10.3 ± 2.2	AV < 15 (L1)
Emulsification time	Visual	95 ± 11.7 s	<120 s
Droplet size (z-average)	DLS	168 ± 9.7 nm	<200 nm
Polydispersity index	DLS	0.38 ± 0.07	<0.4
Drug content	HPLC	92.5 ± 8.7%	90.0–110.0%

**Table 9:** Optimal response and slacks for the WGP approach. Aspiration level is shown as a reference as well as the slacks or deviations (ni and pi). AR: angle of repose; BE: blending efficiency; CR: CLZ recovery.

Response	Alias	Optimal Value	Aspiration Level (*T_i_*)	*n_i_*	*p_i_*
AR	z_1_	38.96	35	0	3.96
BE	z_2_	0.11	0.12	0.00592	0
CR	z_3_	90.23	90	0	0.23

## Data Availability

The original contributions presented in this study are included in the article/Supplementary Material. Further inquiries can be directed to the corresponding author.

## Supplementary Materials

The following supporting information can be downloaded at: https://www.mdpi.com/article/10.3390/pharmaceutics18030305/s1. Figure S1: Representative droplet size distribution plots (DLS) measured by intensity for preliminary SEDDS formulations. (a) Batch 0: Initial surfactant-water system showing large aggregates. (b) Batch 1 and (c) Batch 4: Progressive refinement of the bimodal distribution as the oil/surfactant ratio is adjusted. (d) Batch 5: Optimized ratio using Gelucire^®^ 50/13. (e) Batch 6: Optimized system after replacing Gelucire^®^ 50/13 by Tween^®^ 80, showing a significant shift in the primary population towards the ultra-fine range (<50 nm). Note the logarithmic scale on the x-axis (Size in d.nm) and the intensity percentage on the y-axis. Figure S2: Statistical Analysis of Means (ANOM) for Angle of Repose (z_1_). Figure S3. Statistical Analysis of Means (ANOM) for Blending Efficiency (z_2_). Figure S4. Statistical Analysis of Means (ANOM) for CLZ Recovery (z_3_). Figure S5. 2D contour plots illustrating the effect of formulation variables (F1, F2 and F3) on the Angle of Repose (AR). The remaining factor (F4) maintained fixed its optimum level. Figure S6. 2D contour plots illustrating the effect of formulation variables (F1, F2, F3 and F4) on the Blending Efficiency (BE). Figure S7. Differential Scanning Calorimetry (DSC) thermograms of S-SEDDS components and the optimized formulation. Figure S8. TF-XRD diffractograms and crystallinity data. Table S1: Droplet size distribution of liquid SEDDS formulations (F0–F6) in acidic medium (pH 1.2). Table S2: Multi Linear Regression ANOVA for z_1_. Table S3: Multi Linear Regression ANOVA for z_2_. Table S4: Multi Linear Regression ANOVA for z_3_.

## Abbreviations

The following abbreviations are used in this manuscript:

SSRIs	Selective Serotonin Reuptake Inhibitors
SNRIs	Serotonin-Norepinephrine Reuptake Inhibitors
CLZ	Clonazepam
SEDDS	Self-Emulsifying Drug Delivery System
S-SEDDS	Solid Self-Emulsifying Drug Delivery System
QbD	Quality by Design
D	Desirability function
WGP	Weight Goal Programming
HPLC	High Performance Liquid Chromatographic
PDI	Polydispersity Index
SGF	Simulated Gastric Fluid
DLS	Dynamic Light Scattering
AR	Angle of Repose
BE	Blending Efficiency
CR	Clonazepam Recovery
CQA	Critical Quality Attribute
CV	Coefficient of Variation
QTTP	Quality Target Product Profile
CPP	Critical Process Parameter
CMA	Critical Material Attribute
BBD	Box–Behnken Design
Bcon	Continuous Blending
Bman	Manual Blending
Bint	Blending by Intervals
ANOVA	Analysis of Variance
ANOM	Analysis of Means
DS	Design Space
LTB	Larger-The-Better
STB	Smaller-The-Better
ESEM	Environmental Scanning Electron Microscopy
AV	Acceptance Value
