# Supplementary material for: Advanced Optimization of Clonazepam-Loaded Solid Self-Emulsifying Drug Delivery Systems: Comparison of Weighted Goal Programming and Desirability Function in a Quality by Design Framework
Source: Pharmaceutics. 2026 Feb 28;18(3):305. doi: 10.3390/pharmaceutics18030305 (PMC13029302; doi:10.3390/pharmaceutics18030305)
Supplement: Supplementary file 1 [file pharmaceutics-18-00305-s001.zip › pharmaceutics-4146417-supplementary.pdf]

Supplementary Materials: Advanced Optimization of Clonazepam-Loaded Solid Self-Emulsifying Drug Delivery Systems: Comparison of Weighted Goal Programming and Desirability Function in a Quality by Design Framework

María Luisa González-Rodríguez, Sonia Valverde-Cabeza, Enrique Pérez-Terrón, Antonio María Rabasco and Pedro Luis González-R

The following figures present the representative droplet size distribution reports obtained during the preliminary screening of liquid Self-Emulsifying Drug Delivery Systems (SEDDS). The objective of this screening was to evaluate the spontaneous emulsification capacity of different lipid-surfactant combinations in an acidic environment (pH 1.2), simulating gastric conditions. The analysis was performed using Dynamic Light Scattering (DLS), where the Intensity (Percent) on the y-axis represents the relative amount of light scattered by each population, and the Size (d.nm) on the x-axis indicates the hydrodynamic diameter of the droplets on a logarithmic scale.

Figures S1(a)-S1(e) utilized Gelucire® 50/13 as the primary surfactant. The diagrams show a characteristic bimodal distribution with a significant presence of larger aggregates or droplets (>500 nm). Formula 6 represents the optimized liquid template where Tween® 80 replaced Gelucire® 50/13. The corresponding diagram shows a notable shift of the primary population into the sub-60 nm range, indicating superior interfacial stabilization and a more efficient self-emulsification process. These data provided the experimental basis for selecting the optimized lipid/surfactant ratio for the subsequent solidification process using QbD principles.

(a)

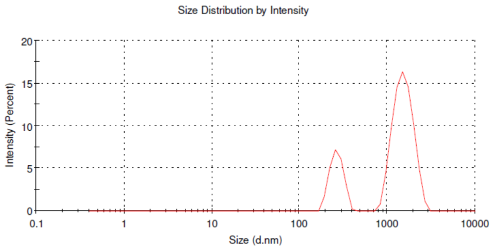

(b)

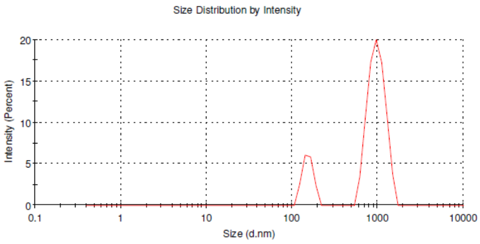

(c)

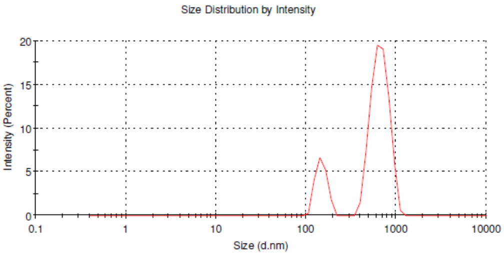

(d)

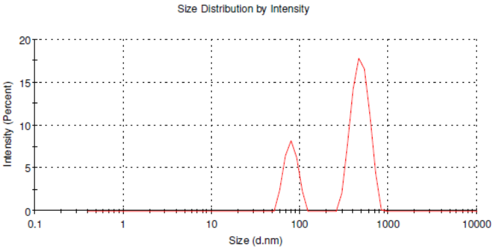

(e)

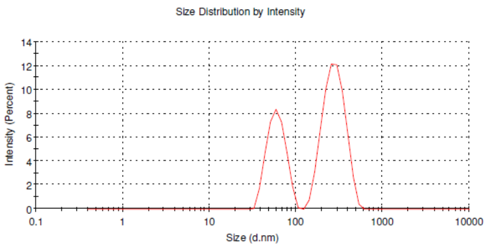

Figure S1. Representative droplet size distribution plots (DLS) measured by intensity for preliminary SEDDS formulations. (a) Formula 0: Initial surfactant-water system showing large aggregates. (b) Formula 1 and (c) Formula 4: Progressive refinement of the bimodal distribution as the oil/surfactant ratio is adjusted. (d) Formula 5: Optimized ratio using Gelucire® 50/13. (e) Formula 6: Optimized system after replacing Gelucire® 50/13 by Tween® 80, showing a significant shift of the primary population towards the ultra-fine range (< 60 nm). .

Table 1. F4) cause a response to deviate significantly from the overall experimental average at a confidence level of $\alpha = 0.1$. Each chart presents the decision limits: the Upper Decision Limit (UDL) and the Lower Decision Limit (LDL). Points falling outside these boundaries indicate a statistically significant effect of that specific factor level on the response.

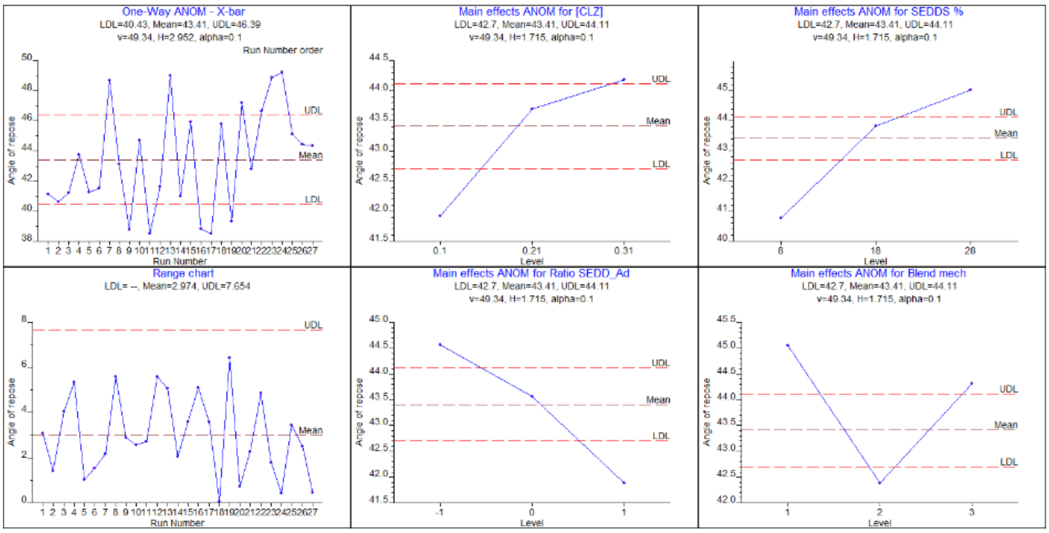

Figure S2. Statistical Analysis of Means (ANOM) for Angle of Repose (z_1). The left panels show the global process stability (X-bar and Range charts), while the right panels illustrate the main effects of each factor relative to the grand mean and decision limits (UDL/LDL).

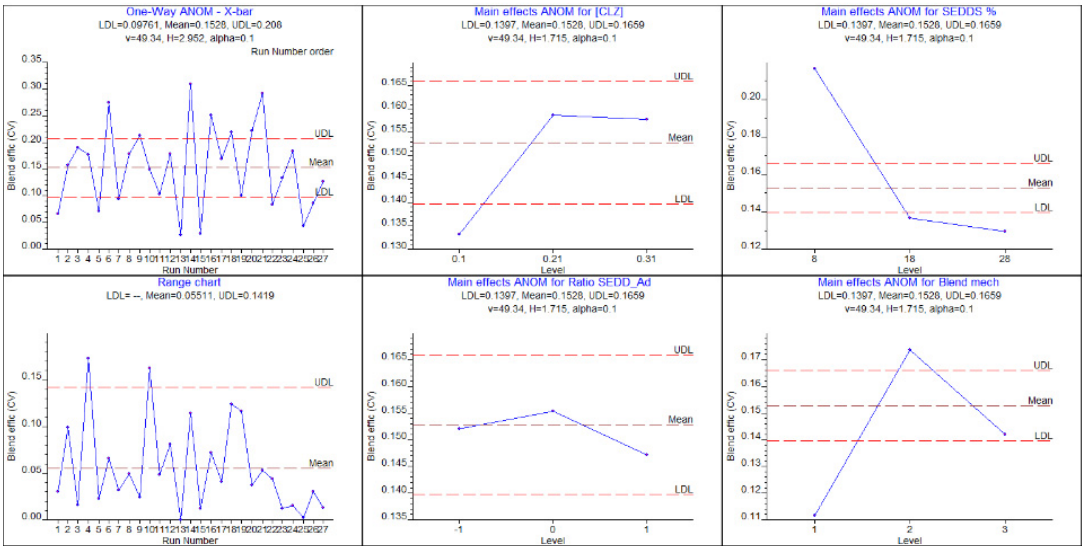

Figure S3. Statistical Analysis of Means (ANOM) for Blending Efficiency (z_2). The left panels show the global process stability (X-bar and Range charts), while the right panels illustrate the main effects of each factor relative to the grand mean and decision limits (UDL/LDL).

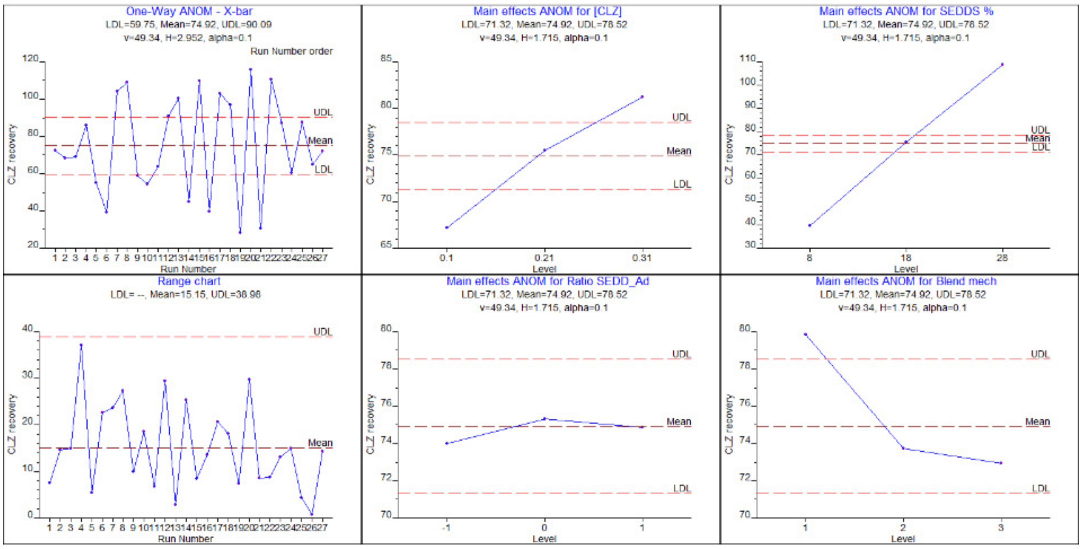

Figure S4. Statistical Analysis of Means (ANOM) for CLZ Recovery (z_3). The left panels show the global process stability (X-bar and Range charts), while the right panels illustrate the main effects of each factor relative to the grand mean and decision limits (UDL/LDL).

This section provides the complete set of 2D contour plots generated from the BBD. While the main manuscript focuses on the interactions between the continuous factors (F1 and F3), these supplementary figures illustrate the interactions involving the discrete (categorical) factors (F2 and F4) across all monitored responses (angle of repose (AR, z_1), blending efficiency (BE, z_2) and CLZ recovery (CR, z_3). Each plot represents the response surface as a function of two variables while maintaining the remaining factors constant at their optimal levels.

(a)

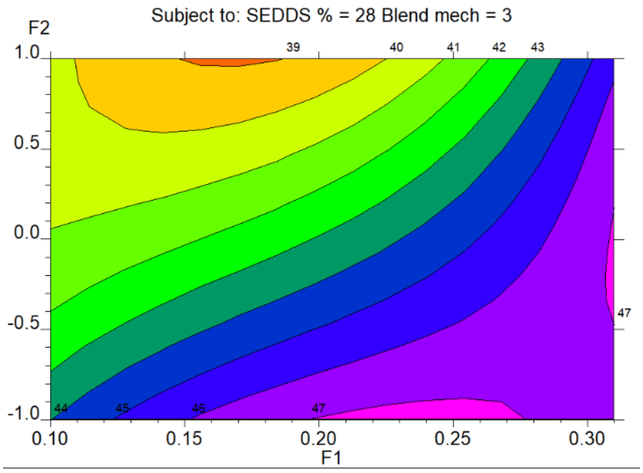

(b)

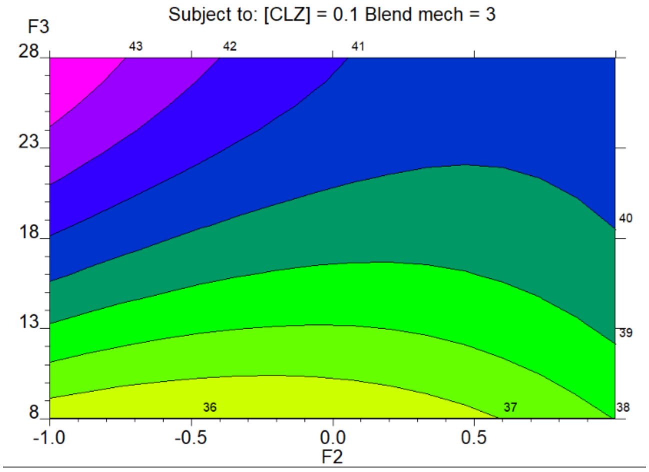

Figure S5. 2D contour plots illustrating the effect of formulation variables (F1, F2 and F3) on the Angle of Repose (AR). The remaining factor (F4) maintained fixed its optimum level. F1: CLZ concentration; F2: SEDDS/Adsorbent ratio; F3: SEDDS percentage; F4: Blending method.

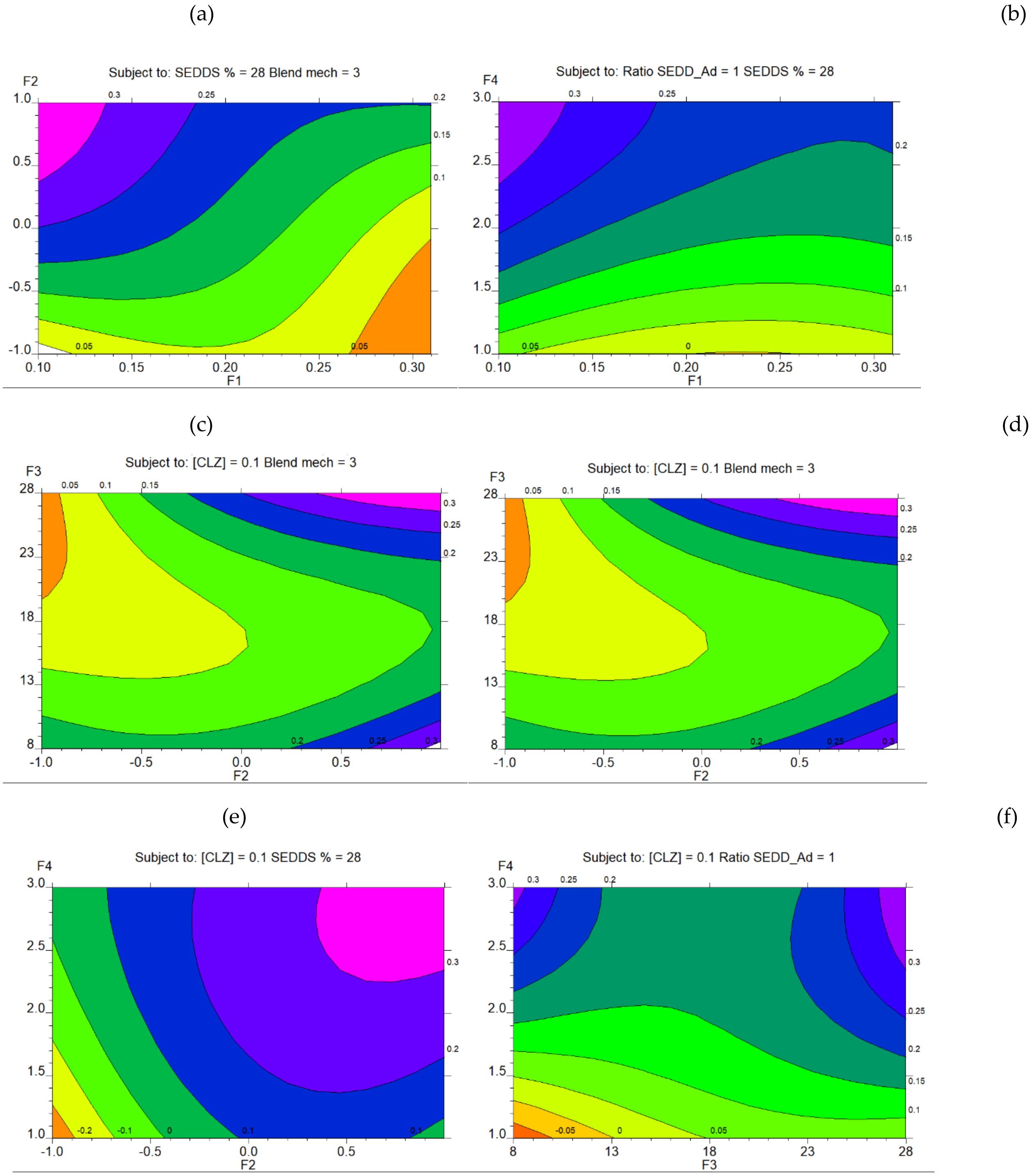

Figure S6. 2D contour plots illustrating the effect of formulation variables (F1, F2, F3 and F4) on the Blending Efficiency (BE). F1: CLZ concentration; F2: SEDDS/Adsorbent ratio; F3: SEDDS percentage; F4: Blending method.

The thermal behavior of pure CLZ, individual carrier (Lactose), the binary (Lactose+Aerosil) and ternary mixtures (Lactose+Aerosil+CLZ), and the optimized S-SEDDS formulation was analyzed using a DSC Q20 differential scanning calorimeter. Samples (approximately 2–5 mg) were accurately weighed and hermetically sealed in aluminum pans. An empty aluminum pan was used as a reference. The analysis was conducted under a continuous nitrogen purge at a flow rate of 100 mL/min to provide an inert atmosphere. The samples were heated from 30 °C to 300 °C at a constant heating rate of 5 °C/min. The overlay of the thermograms was performed using the system software Universal V4.5A TA Instruments 2000 (Figure S7).

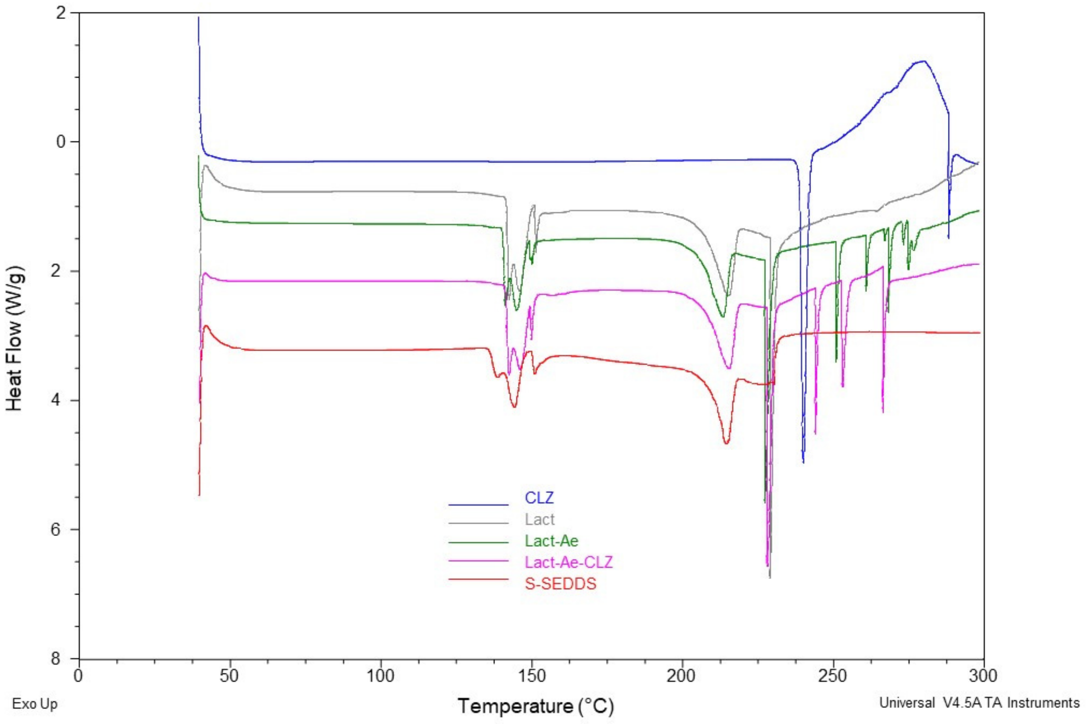

Figure S7. Differential Scanning Calorimetry (DSC) thermograms of S-SEDDS components and the optimized formulation. The analysis includes: pure Clonazepam (CLZ, blue line), showing its characteristic crystalline melting peak at approximately 239 °C; pure Lactose (Lact, grey line); the binary carrier mixture (Lact-Ae, green line); the physical mixture of carriers and drug (Lac-Ae-CLZ, pink line); and the optimized S-SEDDS formulation (SEDDS, red line). The complete disappearance of the CLZ melting endotherm in the S-SEDDS thermogram indicates the successful transformation of the drug into an amorphous state or a molecularly dispersed solid solution within the lipidic-carrier matrix.

The crystalline state of pure CLZ, the solid carriers, and the optimized S-SEDDS formulation was also evaluated by Thin-film X-ray diffraction (TF-XRD) using a Bruker D8 Advance A25 diffractometer in Bragg-Brentano geometry. Cu Kα radiation (λ=0,154 nm) was used as the X-ray source. Data were collected in the 2θ range from 10 ° to 90 ° with a step size of 0.015 ° and a dwell time of 0.1 s per step. This high-resolution setup ensured precise phase identification and crystallinity analysis of the samples.

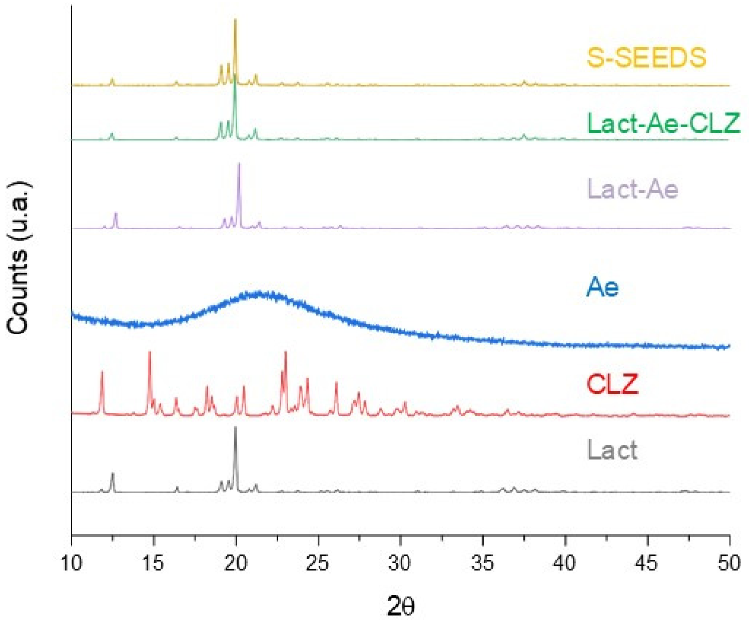

**Sample**	Lact	CLZ	Ae	Lact-Ae	Lact-Ae-CLZ	S-SEDDS
**Crystallinity (%)**	87.9	89.8	41.2	86.6	89.0	80.6

Figure S8. TF-XRD diffractograms and crystallinity data. The patterns represent: Lactose (Lact), pure Clonazepam (CLZ), Aerosil® 200 (Ae), the carrier mixture (Lact-Ae), the drug-carrier physical mixture (Lact-Ae-CLZ), and the optimized S-SEDDS. The table highlights the reduction in crystallinity in the S-SEDDS formulation relative to the crystalline API and its physical mixtures.

The percentage of intensity for each population was recorded to evaluate the homogeneity of the self-emulsification process. As shown in Table S1, the transition from Gelucire® 50/13 (Formula 5) to Tween® 80 (Formula 6) not only reduced the absolute droplet size of both populations but also increased the prevalence of the finest nanometric fraction (46.67±10.64 nm).

Table S1. Droplet size distribution of liquid SEDDS formulations (F0–F6) in acidic medium (pH 1.2).

Batch	Oil %	S/CoS Ratio	Surfactant Type	Mean Size 1 (nm) ± SD	Mean % Intensity 1	Mean Size 2 (nm) ± SD	Mean % Intensity 2	PDI
0	0	97	Gelucire	1425.5±706.7	77.5±14.0	432.6±317.4	22.5±14.0	0.42±0.18
1	17	80	Gelucire	940.1±188.8	82.5±4.7	172.2±37.2	17.5±4.7	0.50±0.07
2	25	72	Gelucire	810.1±80.1	84.6±2.2	158.2± 25.6	16.5±4.1	0.49±0.02
3	35	62	Gelucire	756.5±33.4	83.6±4.1	141.3±20.7	20.1±3.6	0.47±0.04
4	45	52	Gelucire	656.3±11.3	80.2±3.5	132.1±18.9	15.4±2.2	0.42±0.02
5	55	42	Gelucire	471.1±75.5	74.3±3.3	113.3±33.0	25.6±3.3	0.42±0.02
6	45	52	Tween 80	262.2±20.4	71.8±6.9	46.7±10.6	28.2±6.9	0.40±0.02

The following ANOVA tables summarize the contribution of individual factors and their interactions to the variation in each response variable. The Sum of Squares (SS) quantifies the amount of variance explained by each term in the model (higher values indicate greater explanatory power). The Degrees of Freedom (df) correspond to the number of independent values used to estimate the associated effect, typically 1 for individual predictors. The F-statistic assesses the strength of each effect relative to the model's residual variance, computed as the ratio of the mean square of the effect to the mean square of the residuals. The p-value tests the null hypothesis that the corresponding term has no effect; values below a conventional threshold (e.g., 0.05) indicate statistical significance. Statistically significant terms are marked with an asterisk ($*$), highlighting effects with meaningful impact on the response. The Residual row represents the unexplained variance and forms the baseline for F-statistic computation.

Table S2. Multi Linear Regression ANOVA for z_1.

Factor/Interaction	SS	df	F	p-value
F_1	41.367	1	6.255	0.015*
F_2	123.533	1	18.678	0.000*
F_3	70.452	1	10.652	0.002*
F_4	0.297	1	0.045	0.833
$F_1 \times F_2$	6.795	1	1.027	0.315
$F_1 \times F_3$	12.221	1	1.848	0.179
$F_2 \times F_3$	20.593	1	3.114	0.083
$F_1 \times F_4$	138.312	1	20.912	0.000*
$F_2 \times F_4$	0.837	1	0.127	0.723
$F_3 \times F_4$	25.521	1	3.859	0.054
$F_1 \times F_1$	2.001	1	0.303	0.584
$F_3 \times F_3$	5.655	1	0.855	0.359
$F_4 \times F_4$	80.479	1	12.168	0.001*
$F_1 \times F_2 \times F_2$	6.521	1	0.986	0.325
$F_1 \times F_1 \times F_2$	61.957	1	9.368	0.003*
$F_1 \times F_3 \times F_3$	7.763	1	1.174	0.283
$F_1 \times F_1 \times F_3$	11.794	1	1.783	0.187
$F_3 \times F_3 \times F_4$	16.579	1	2.507	0.118
Residual	410.060	62		

Table S3. Multi Linear Regression ANOVA for z_2.

Factor/Interaction	SS	df	F	p-value
F_1	0.008	1	8.556	0.005*
F_2	0.026	1	29.153	0.000*
F_3	0.010	1	10.817	0.002*
F_4	0.047	1	52.151	0.000*
$F_1 \times F_2$	0.018	1	20.404	0.000*
$F_1 \times F_3$	0.081	1	89.418	0.000*
$F_2 \times F_3$	0.029	1	32.452	0.000*
$F_1 \times F_4$	0.002	1	2.384	0.128
$F_3 \times F_4$	0.011	1	11.591	0.001*
$F_1 \times F_1$	0.005	1	5.238	0.026*
$F_2 \times F_2$	0.005	1	5.494	0.022*
$F_3 \times F_3$	0.009	1	10.231	0.002*
$F_4 \times F_4$	0.024	1	26.308	0.000*
$F_1 \times F_2 \times F_2$	0.014	1	15.152	0.000*
$F_1 \times F_1 \times F_2$	0.017	1	18.485	0.000*
$F_1 \times F_3 \times F_3$	0.009	1	9.858	0.003*
$F_2 \times F_3 \times F_3$	0.027	1	29.801	0.000*
$F_2 \times F_2 \times F_3$	0.040	1	43.627	0.000*
$F_2 \times F_2 \times F_4$	0.044	1	48.788	0.000*
$F_3 \times F_3 \times F_4$	0.109	1	120.392	0.000*
$Residual$	0.054	60		

Table S4. Multi Linear Regression ANOVA for z_3.

Factor/Interaction	SS	df	F	p-value
F_1	1773.5230	1	10.185	0.002*
F_3	43077.988	1	247.381	0.000*
F_4	428.386	1	2.460	0.120
$F_1 \times F_1$	52.361	1	0.301	0.585
$F_3 \times F_3$	53.468	1	0.307	0.581
$Residual$	13060.190	75		
